# Supplementary material for: Dependency of EGFR activation in vanadium-based sensitization to oncolytic virotherapy
Source: Mol Ther Oncolytics. 2022 Apr 19;25:146–59. doi: 10.1016/j.omto.2022.04.004 (PMC9065483; doi:10.1016/j.omto.2022.04.004)
Supplement: Document S1. Figures S1–S19 and Tables S1 and S2 [file mmc1.pdf]

OMTO, Volume *25*

Supplemental information

Dependency of EGFR activation in vanadium-based sensitization to oncolytic virotherapy

Boaz Wong, Anabel Bergeron, Nouf Alluqmani, Glib Maznyi, Andrew Chen, Rozanne Arulanandam, and Jean-Simon Diallo

Table S1: Key Resources Table

REAGENT or RESOURCE	SOURCE	IDENTIFIER
Antibodies		
Phosphorylated STAT1 (Tyr701), rabbit	Cell Signaling Technology	7649
STAT1, rabbit	Cell Signaling Technology	9172
Phosphorylated STAT2 (Tyr690), rabbit	Cell Signaling Technology	88410
STAT2, rabbit	Cell Signaling Technology	72604
Phosphorylated EGFR (Tyr1068), rabbit	Cell Signaling Technology	2234
EGFR, rabbit	Cell Signaling Technology	4267
Phosphorylated NF-κB/p65 (Ser536), rabbit	Cell Signaling Technology	3033
NF-κB/p65, rabbit	Cell Signaling Technology	8242
IκB-α (Amino-terminal antigen), mouse	Cell Signaling Technology	4814
Lamin B1, rabbit	Cell Signaling Technology	12586
α-tubulin, mouse	Santa Cruz	sc-8035
β-actin, rabbit	Cell Signaling Technology	4970
hFAB™ Rhodamine Anti-Actin	Bio-Rad	12004163
hFAB™ Rhodamine Anti-GAPDH	Bio-Rad	12004168
Anti-rabbit HRP Secondary Antibody	New England Biolabs	7074
Anti-rabbit IgG (H+L) Alexa Fluor® 555	Cell Signaling Technology	4413S
Bacterial and virus strains		
VSVΔ51-Green fluorescent protein (GFP)	Dr. Jean-Simon Diallo	
VSVΔ51ΔG-GFP	Dr. Jean-Simon Diallo	
VSVΔ51-Firefly luciferase (FLuc)	Dr. Jean-Simon Diallo	
Measles virus, Schwartz strain - GFP	Dr. Guy Ungerechts	
Chemicals, peptides, and recombinant proteins		
Gefitinib	BioVision	1589
Erlotinib	BioVision	2048
UO126	Sigma-Aldrich	662005
IMD-0354	Sigma-Aldrich	I3159

SC-514	Sigma-Aldrich	SML0557
Human EGF	R&D Systems	236-EG
Human EGFR (cetuximab) antibody	InvivoGen	hegfr-mab1
Human IFNβ	PBL Assay Science	11415-1
Critical commercial assays		
Human CXCL10/IP-10 DuoSet Assay kit	R&D Systems	DY266
Deposited Data		
All uncropped Western blot images and raw data	Mendeley Data	doi:10.17632/zzpvtnmdw7.1
Experimental models: Cell lines		
786-0, human renal cell carcinoma	ATCC	CRL-1932
CT26WT, murine colon carcinoma	ATCC	CRL-2638
Vero, African green monkey renal cells	ATCC	CRL-81
TC-1, murine lung carcinoma	Dr. Guy Ungerechts	
Experimental models: Organisms/strains		
Female Balb/c mice	Charles River Laboratories	
Oligonucleotides		
Please see supplementary table S2 for oligonucleotides	Thermo Scientific	
Software and algorithms		
Prism 9.0	GraphPad	

Table S2: List of primers used in this study.

**Model**	**Gene**	**Forward primer (5' to 3')**	**Reverse primer (5' to 3')**
VSV	M	ATACTCAGATGTGGCAGCCG	GATCTGCCAATACCGCTGGA
	N	CATGTCACTGCAAGGCCTAAGA	GGCAGTATCGTGAATTCGATGC-3
Human	STAT1	ATGGCAGTCTGGCGGCTGAATT	CCAAACCAGGCTGGCACAATTG
	STAT2	CAGGTCACAGAGTTGCTACAGC	CGGTGAACTTGCTGCCAGTCTT
	GAPDH	ACAGTCAGCCGCATCTTCTT	GTTAAAAGCAGCCCTGGTGA
	IFNβ	CATTACCTGAAGGCCAAGGA	CAGCATCTGCTGGTTGAAGA
	IL-1b	CCACAGACCTTCCAGGAGAATG	GTGCAGTTCAGTGATCGTACAGG
	TNF-α	GCTGCACTTTGGAGTGATCG	GAGGGTTTGCTACAACATGGG
	CCL5	GCAGTCGTCCACAGGTCAAG	TCTTCTCTGGGTTGGCACAC
	IL-6	ACCCCCAATAAATATAGGACTGGA	GAAGGCGCTTGTGGAGAAGG
	CXCL9	AGTGCAAGGAACCCCAGTAG	AGGGCTTGGGGCAAATTGTT
	CXCL10	CTGAGCCTACAGCAGAGGAAC	AGGTACTCCTTGAATGCCACTT

	MX2	GAACGTGCAGCGAGCTTGTC	AAGGCTTGTGGGCCTTAGAC
	IFITM1	CCGTGAAGTCTAGGGACAG G	GGTAGACTGTCACAGAGCCG
Mouse	GAPDH	CATCACTGCCACCCAGAAGA CTG	ATGCCAGTGAGCTTCCCGTT CAG
	CXCL9	CAGTGTGGAGTTCGAGGAAC C	TTTGTTGCAATTGGGGCTTG G
	CXCL10	ATCATCCCTGCGAGCCTATC CT	GACCTTTTTTGGCTAAACGC TTTC

S1

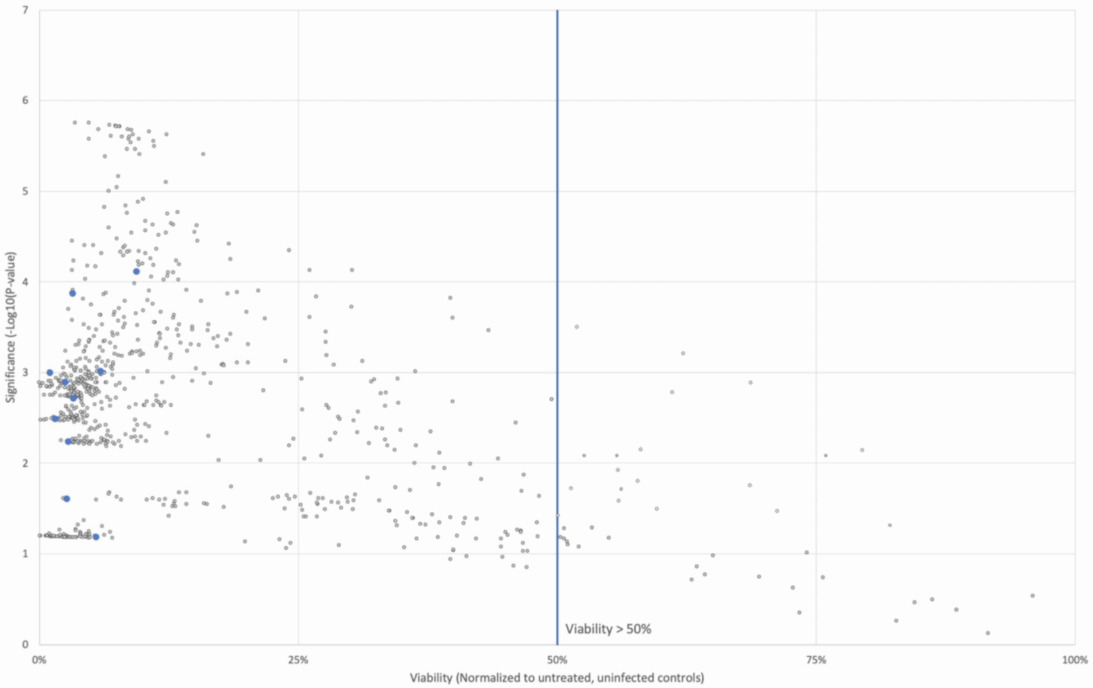

Significance (-Log10(P-value))
Viability (Normalized to untreated, uninfected controls)
Viability > 50%
0%
25%
50%
75%
100%
0
1
2
3
4
5
6
7

S2

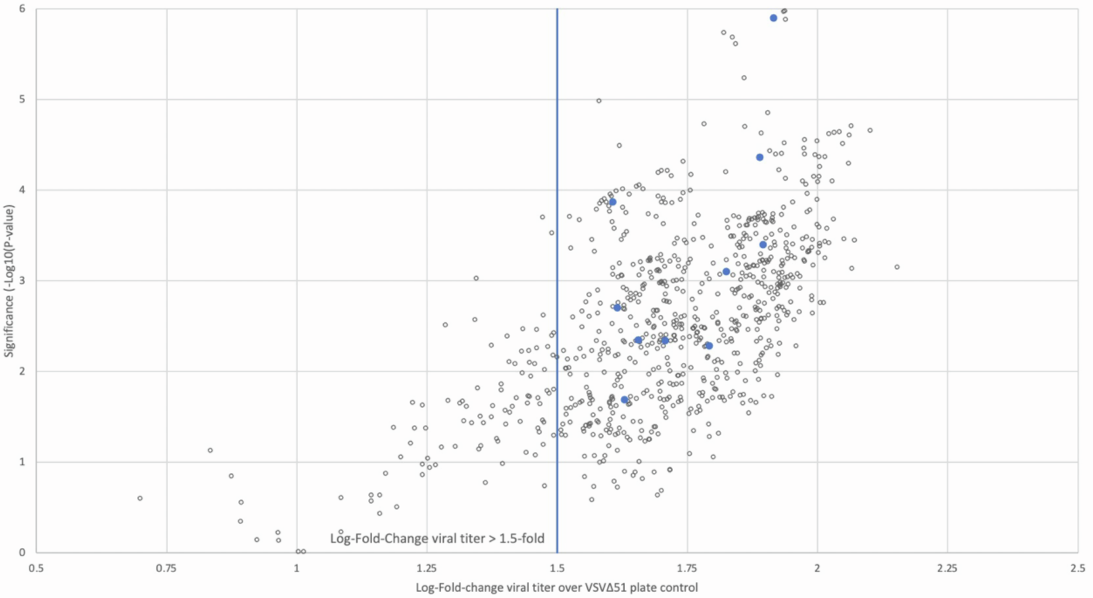

Significance (-Log10(P-value))
Log-Fold-change viral titer over VSVΔ51 plate control
Log-Fold-Change viral titer > 1.5-fold
0.5
0.75
1
1.25
1.5
1.75
2
2.25
2.5
0
1
2
3
4
5
6

Figure S1: Scatter plot of reverse kinase screen viability. 786-0 cells were pre-treated with a drug library of kinase inhibitors (1µM) and vanadate (125µM) for four hours and subsequently infected with VSVΔ51-FLuc at a MOI of 0.1. Vanadate controls are shown in blue. 48 hours post infection (hpi), the metabolic activity (surrogate for viability) was measured using resazurin (Alamar blue) assay. Values were blank-controlled then normalized against infected, untreated 786-0 (mock-infected) controls. An unpaired t-test assuming unequal variances was performed, the y-axis represents the calculated $-\text{Log}_{10}$(P-value) (n=2-3).

Figure S2: Scatter plot of reverse kinase screen vial titer. 48 hours post infection (hpi), the viral titer was determined using high-throughput virus assay. Values were normalized against infected, untreated 786-0 (mock-infected) controls. An unpaired t-test assuming unequal variances was performed, the y-axis represents the calculated $-\text{Log}_{10}$(P-value) (n=2-3).

S3

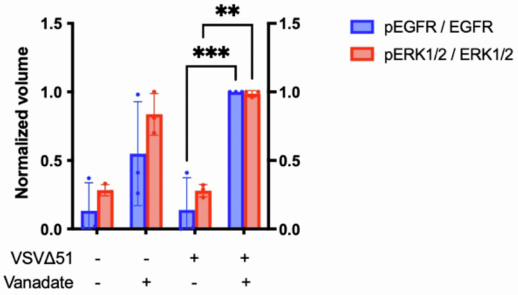
pEGFR / EGFR
pERK1/2 / ERK1/2
Normalized volume
**
***
VSVΔ51 - - + +
Vanadate - + - +

S4

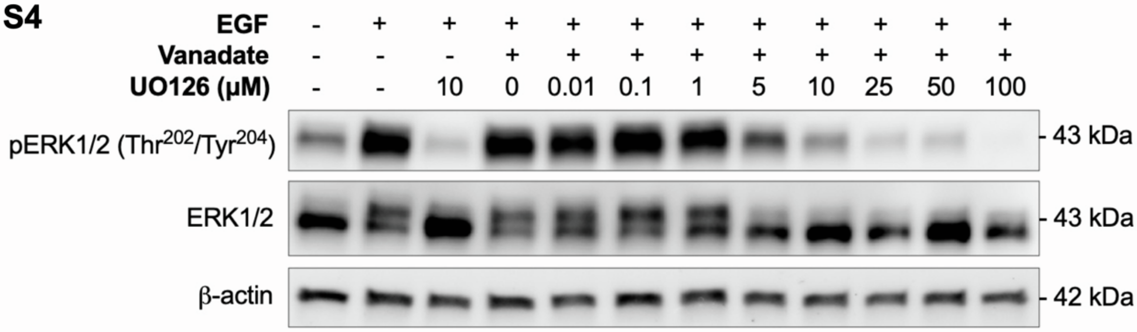
EGF - + + + + + + + + + + +
Vanadate - - - + + + + + + + + +
UO126 (μM) - - 10 0 0.01 0.1 1 5 10 25 50 100
pERK1/2 (Thr202/Tyr204)
ERK1/2
β-actin
43 kDa
43 kDa
42 kDa

S5

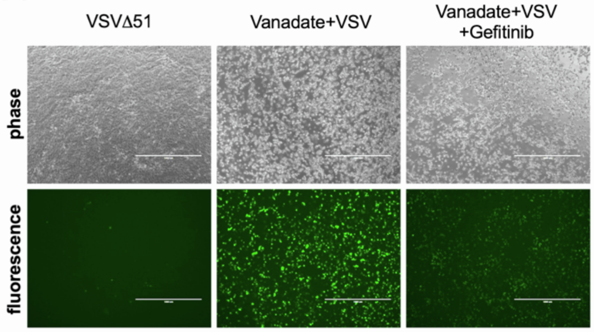
VSVΔ51
Vanadate+VSV
Vanadate+VSV +Gefitinib
phase
fluorescence

S6

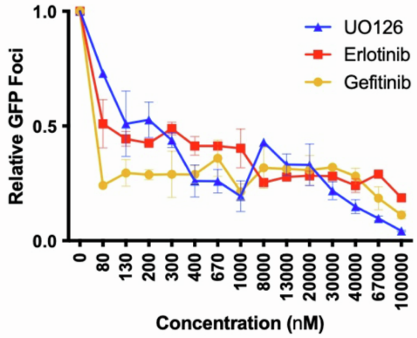
UO126
Erlotinib
Gefitinib
Relative GFP Foci
Concentration (nM)

S7

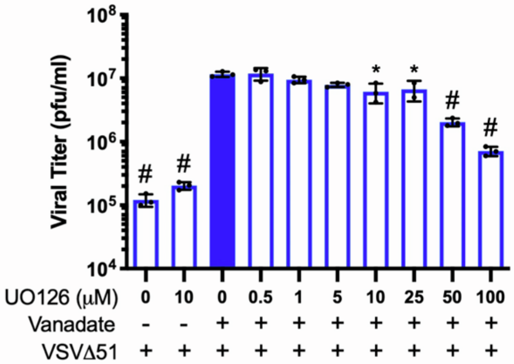
Viral Titer (pfu/ml)
UO126 (μM) 0 10 0 0.5 1 5 10 25 50 100
Vanadate - - + + + + + + + +
VSVΔ51 + + + + + + + + + +

Figure S3: pEGFR:EGFR and pERK1/2:ERK1/2 volume densitometry ratios corresponding to Figure 1D (n=3, mean ± SD).

Figure S4: Human 786-0 renal carcinoma cells were treated pre-treated with or without vanadate (150 μM) and varying concentrations of UO126 (0 – 100μM) as indicated for four hours. Cells were then treated with 10ng/mL EGF for 10 minutes and lysates were probed by western blot for phosphorylated ERK1/2, total ERK1/2 and β-actin.

Figure S5: Human 786-0 cells were pretreated ± vanadate (150μM) ± gefitinib (10μM) for 4 hours, then consequently infected with VSVΔ51-GFP (MOI 0.01). Phase and fluorescence images were taken 24 hours post infection (hpi); scale bar = 1000μm.

Figure S6: Count of GFP foci were obtained from Figure 2B and plotted relative to the vanadate + VSVΔ51 condition without kinase inhibitor (n=2, mean ± SEM).

Figure S7: Human 786-0 cells were pretreated ± vanadate (150μM) ± UO126 (0 – 100μM) for 4 hours, then consequently infected with VSVΔ51-GFP (MOI 0.01). Supernatants were collected 24hpi and titered by viral plaque assay (n=3, mean ± SD; *$P<0.05$, $^{\#}P<0.0001$; one-way ANOVA compared to the infected VSVΔ51 + vanadate only condition as indicated by the filled bar).

S8

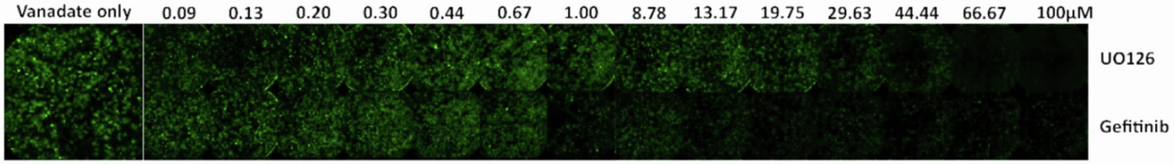
Vanadate only
0.09
0.13
0.20
0.30
0.44
0.67
1.00
8.78
13.17
19.75
29.63
44.44
66.67
100μM
UO126
Gefitinib

S9

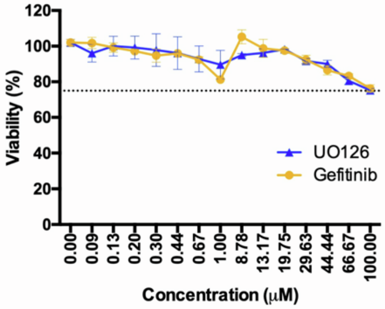
Viability (%)
120
100
80
60
40
20
0
0.00
0.09
0.13
0.20
0.30
0.44
0.67
1.00
8.78
13.17
19.75
29.63
44.44
66.67
100.00
Concentration (μM)
UO126
Gefitinib

S10

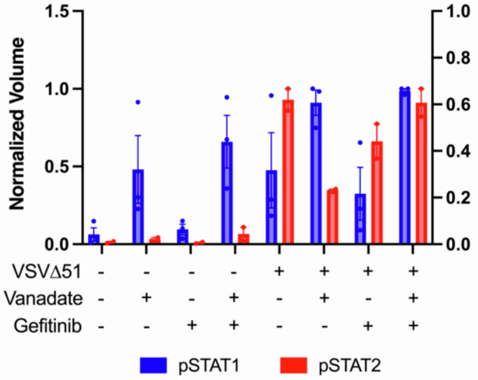
Normalized Volume
1.5
1.0
0.5
0.0
1.0
0.8
0.6
0.4
0.2
0.0
VSVΔ51 - - - - + + + +
Vanadate - + - + - + - +
Gefitinib - - + + - - + +
pSTAT1
pSTAT2

S11

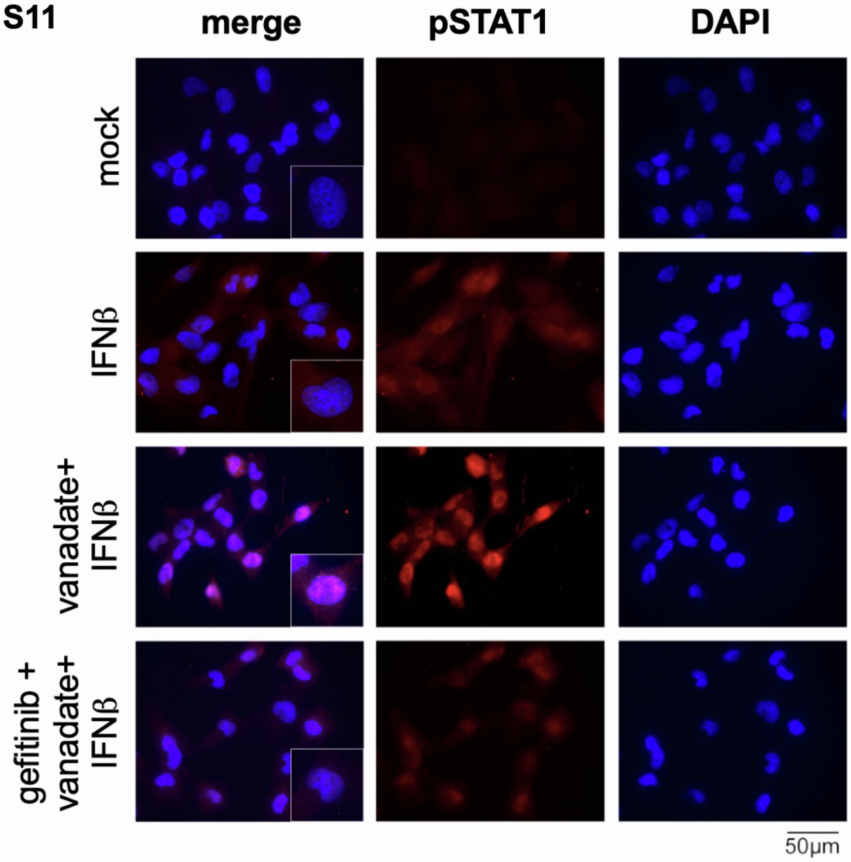
merge
pSTAT1
DAPI
mock
IFNβ
vanadate+ IFNβ
gefitinib + vanadate+ IFNβ
50μm

Figure S8: Human renal 786-0 cancer cells were simultaneously treated with varying concentrations of gefitinib or UO126 (0 - 100 µM) and vanadate (150 µM). Four hours later, cells were infected with measles virus expressing GFP (MOI 0.3). Representative fluorescent images were taken 24 hours post infection (hpi).

Figure S9: Cell viability was measured by Alamar blue assay and compared relative to the vanadate + VSVΔ51 condition without kinase inhibitor (n=2, mean ± SEM). Dotted line represents 75% viability.

Figure S10: Volume densitometry of pSTAT1 and pSTAT2 levels from Figure 3A (n=3, mean ± SD).

Figure S11: Representative immunofluorescence images corresponding to Figure 3D. Scale bar = 50µm.

S12

TF	*Pval*	*Eval*	∩	#TG	RC (%)
STAT1	< 1.0E-06	< 1.0E-06	8	25	0
NFKB1	4.00E-05	1.68E-03	7	63	0

S13

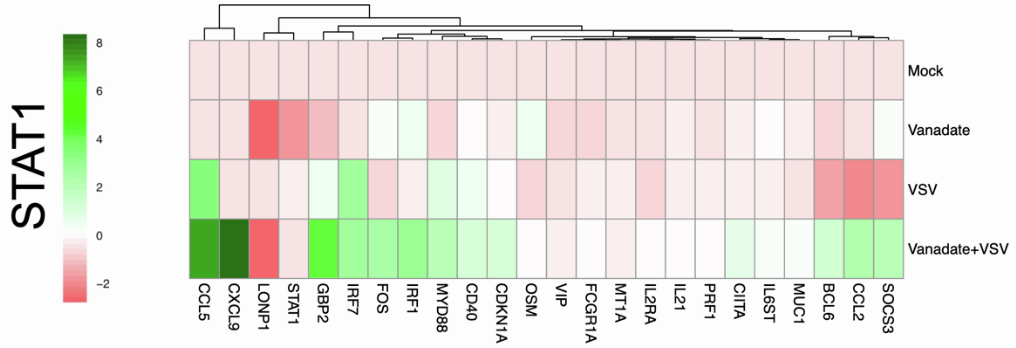

S14

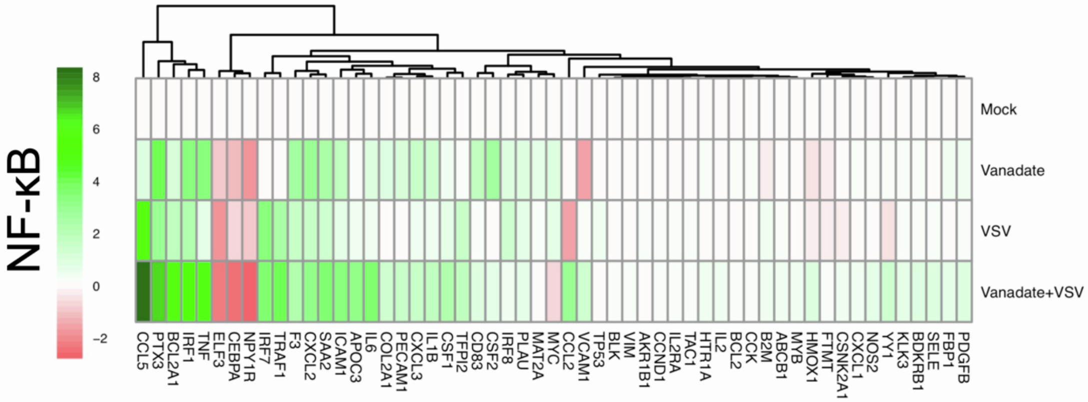

S15

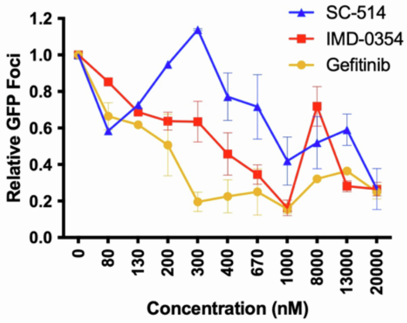

S16

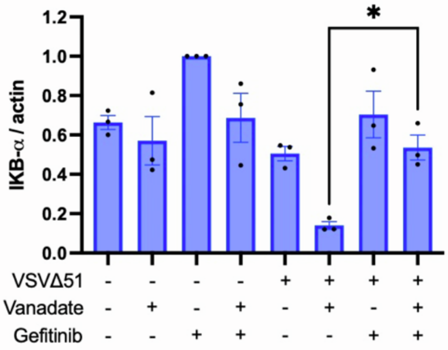

Figure S12: Table listing STAT1 and NF-κB transcription factors from the TFactS *in silico* analysis: *Pval* is P-value, *Eval* is E-value, ∩ is the number of genes common between TF target genes and the query list, #TG is number of target genes for the corresponding TF and RC(%) is random control percentage which is a non-parametric control of false positives. Refer to published paper for more details on these parameters [29].

Figure S13: Heatmap of genes downstream to STAT1 transcription factor on 786-0 cells treated ± vanadate (150μM) and ±VSVΔ51 infection (MOI 0.01).

Figure S14: Heatmap of genes downstream to NF-κB transcription factor on 786-0 cells treated ± vanadate (150μM) and ±VSVΔ51 infection (MOI 0.01).

Figure S15: Count of GFP foci were obtained from Figure 4B and plotted relative to the vanadate + VSVΔ51 condition without kinase inhibitor (n=2, mean ± SEM).

Figure S16: Volume densitometry of IκB-α levels from Figure 4E (n=3, mean ± SD, *P<0.05 by one-way ANOVA).

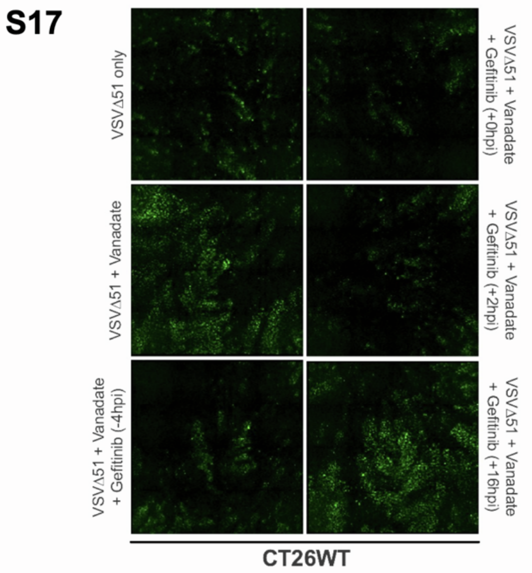

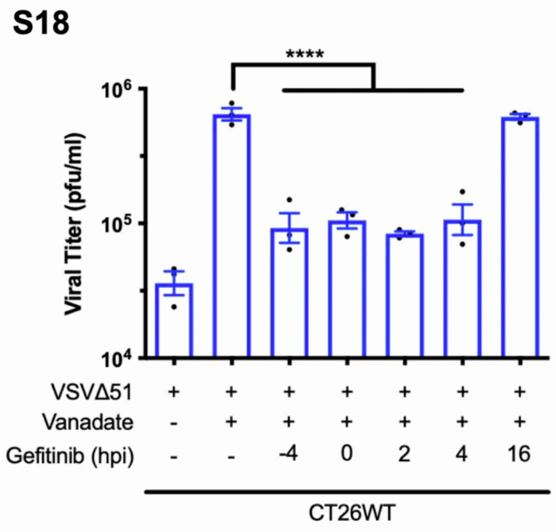

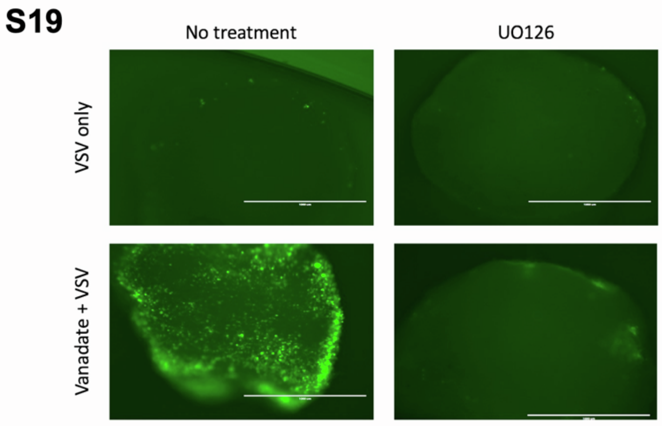

Figure S17: Mouse CT26WT colon carcinoma cells were pretreated ± vanadate (150µM) for 4 hours ± gefitinib (10µM) at -4, 0.5, 1, 2, 4 and 16 hours post infection (hpi), then consequently infected with VSVΔ51-GFP (MOI 0.01). Representative images are shown.

Figure S18: Supernatant from Figure S16 was collected 24hpi and titered by plaque assay (n=3, mean ± SD; ****P<0.0001 by one-way ANOVA).

Figure S19: *Ex vivo* mouse CT26WT tumors implanted subcutaneously in Balb/c mice were pretreated ± vanadate (150µM) ± UO126 (20µM) for 4 hours, then consequently infected with VSVΔ51-GFP (3e4 pfu/core). Representative fluorescence images were taken 24 hours post infection. Scale bar = 1000 µm.
