# Supplementary material for: Dependency of EGFR activation in vanadium-based sensitization to oncolytic virotherapy
Source: Mol Ther Oncolytics. 2022 Apr 19;25:146–59. doi: 10.1016/j.omto.2022.04.004 (PMC9065483; doi:10.1016/j.omto.2022.04.004)
Supplement: Document S2. Article plus supplemental information [file mmc2.pdf]

Molecular Therapy
Oncolytics
Original Article

Dependency of EGFR activation in vanadium-based sensitization to oncolytic virotherapy

Boaz Wong,[1,2] Anabel Bergeron,[1,2] Nouf Alluqmani,[1,2] Glib Maznyi,[1] Andrew Chen,[1] Rozanne Arulanandam,[1] and Jean-Simon Diallo[1,2]

[1]Centre for Innovative Cancer Research, Ottawa Hospital Research Institute, 501 Smyth Road, Ottawa, ON K1H 8L6 Canada; [2]Department of Biochemistry, Microbiology and Immunology, Faculty of Medicine, University of Ottawa, Ottawa, ON K1H 8M5, Canada

Oncolytic virotherapy is a clinically validated approach to treat cancers such as melanoma; however, tumor resistance to virus makes its efficacy variable. Compounds such as sodium orthovanadate (vanadate) can overcome viral resistance and synergize with RNA-based oncolytic viruses. In this study, we explored the basis of vanadate mode of action and identified key cellular components in vanadate's oncolytic virus-enhancing mechanism using a high-throughput kinase inhibitor screen. We found that several kinase inhibitors affecting signaling downstream of the epidermal growth factor receptor (EGFR) pathway abrogated the oncolytic virus-enhancing effects of vanadate. EGFR pathway inhibitors such as gefitinib negated vanadate-associated changes in the phosphorylation and localization of STAT1/2 as well as NF-κB signaling. Moreover, gefitinib treatment could abrogate the viral sensitizing response of vanadium compounds *in vivo*. Together, we demonstrate that EGFR signaling plays an integral role in vanadium viral sensitization and that pharmacological EGFR blockade can counteract vanadium/oncolytic virus combination therapy.

INTRODUCTION

Oncolytic virotherapy (OV) is a class of biotherapeutics that uses viruses to selectively infect, replicate within, and lyse tumor cells while triggering a lasting anti-tumor immune response.[1–3] OV therapy can result in lasting cures with an improved long-term side effect profile when compared with conventional chemotherapy modalities.[4] Over the past several years, oncolytic virotherapy has made tremendous progress toward clinical use. Hundreds of different OV products using different viral platforms and genetic payloads are currently in clinical trial.[5] Notably, the clinical regulatory approval of talimogene laherparepvec (T-VEC), a genetically engineered herpes simplex virus (HSV-1) for the treatment of melanoma, has unveiled the potential of OVs as a staple cancer therapeutic in years to come.[6,7]

Unfortunately, clinical developments of OV monotherapies have stalled in part due to frequent tumor resistance to infection.[8] A key mediator of the resistance to OV therapy is the type 1 interferon (IFN-1) response that induces antiviral gene expression and inhibits viral spread.[9,10] To combat this, small molecules that transiently modify the interferon response have been investigated in order to recover oncolytic efficacy.[11–14] Our group has identified sodium orthovanadate (vanadate) and other vanadium-based compounds as being capable not only of attenuating the antiviral IFN-1 response, but also simultaneously increasing proinflammatory activity through type II interferon (IFN-2) signals when co-administrated with the oncolytic vesicular stomatitis virus (VSVΔ51).[15,16] In addition to reducing IFN-1 responses, vanadate treatment increases virus-induced proinflammatory cytokines including interferon-beta (IFN-β), tissue necrosis factor alpha (TNF-α), and interleukin 6 (IL-6). We have previously shown that the effect of vanadate on the IFN-1 and IFN-2 response correlates with the accumulation of phosphorylated STAT1 (signal transducer and activation of transcription) transcription factor in the nucleus and reduced STAT2 expression/activation.[15] However, the exact cell signaling cascade that gives rise to this effect currently remains unknown.

Vanadium-based compounds confer their biological effects through pan-inhibition of protein tyrosine phosphatases (PTP) in part through competition with phosphate.[17,18] PTPs normally elicit their effects through the de-phosphorylation of substrates, counteracting the action of kinases that phosphorylate these same substrates. Given this homeostasis, vanadium maintains the phosphorylated state of multiple cellular substrates, typically resulting in the persistent activation of their downstream signal transduction pathways. By shifting homeostasis toward the activity of kinases, inhibiting PTPs using vanadium therefore leads to multiple effects, including drug resistance reversal, inhibition of cellular proliferation, and, of particular interest, immunomodulation.[19] The objective of the current study was to further elucidate the mechanism of action by which vanadium-based compounds confer viral sensitizing properties with intentions to further understand OV resistance patterns and to inform the design of improved viral sensitization strategies. As a strategy to achieve greater mechanistic insight, we rationalized that systematically testing the impact of kinase inhibition on vanadate's OV-enhancing effect might allow us to identify key shared

Received 13 November 2021; accepted 14 April 2022;
https://doi.org/10.1016/j.omto.2022.04.004.

Correspondence: Jean-Simon Diallo, PhD, Centre for Innovative Cancer Research, Ottawa Hospital Research Institute, 501 Smyth Road, Ottawa, ON K1H 8L6 Canada.
E-mail: jsdiallo@ohri.ca

 © 2022 The Authors.
This is an open access article under the CC BY license (http://creativecommons.org/licenses/by/4.0/).

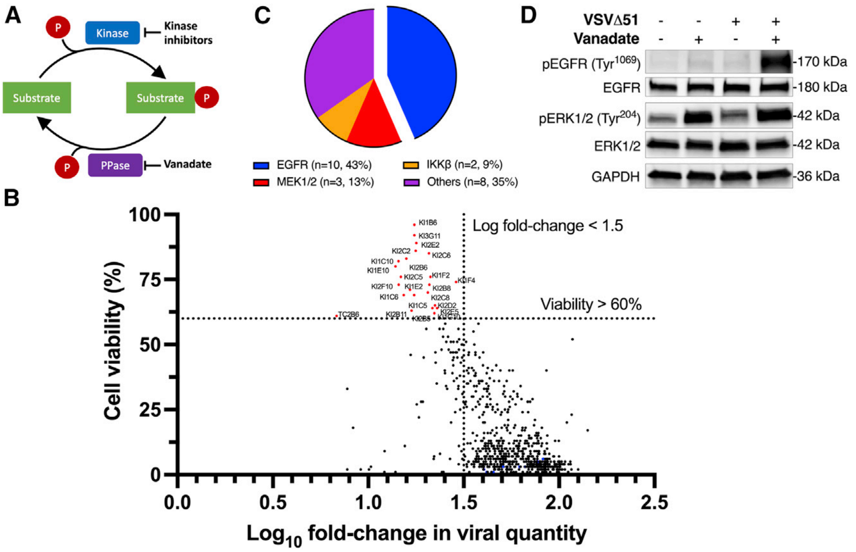

Figure 1. Identification of EGFR signaling to vanadate viral sensitization

(A) Schematic depicting kinase-phosphatase homeostasis as a mechanistic principle for understanding vanadate-mediated viral sensitization, PPase = phosphatase. (B) Human renal carcinoma 786-0 cells were pre-treated for 4 h with vanadate (125 μM) and kinase inhibitors (1 μM), then subsequently infected with VSVΔ51-FLuc (MOI 0.1). Viability was assessed 48 h by resazurin (Alamar blue) assay after infection and measures were normalized against VSVΔ51 plate controls. Viral titers were quantified 48 hpi by high-throughput titration. Plot shows cell viability against log fold-change in viral titers of all compounds. Compiled viability and viral titer data highlight a subset of 23 kinase inhibitors that simultaneously prevent vanadate's oncolysis and viral replication, depicted in red (Table 1). Vanadate + VSVΔ51 plate controls are depicted in blue (n = 2–3). Dotted lines represent viability and titer thresholds (60% and 1.5 fold-change, respectively). (C) Pie chart representing relative proportions of molecular targets of short-listed kinase inhibitors identified in (B). (D) The 786-0 cells were pre-treated ± vanadate (150 μM), then infected with VSVΔ51 (MOI 0.1). Cell lysates were collected 24 hpi and probed for phosphorylated EGFR, total EGFR, phosphorylated ERK1/2, total ERK1/2, and GAPDH by western blot.

PTP/kinase target substrates implicated in mediating the enhancing effects of vanadate.

RESULTS

High-throughput kinase inhibitor screen identifies importance of epidermal growth factor receptor in viral sensitization

We hypothesized that, as a pan-PTP inhibitor, vanadate might potentiate virotherapy by promoting the phosphorylation state of one or more proteins relevant in modulating the response to IFN-1. Inhibition of related kinases would thus reverse the required phosphorylation states for IFN-1 inhibition (Figure 1A). To pinpoint these relevant kinases, 800 small molecules (kinase inhibitors and various tool compounds) were tested using a reverse screen strategy. Briefly, 786-0 cells, a human renal adenocarcinoma cell line naturally resistant to VSVΔ51 infection,[11] were treated with an optimized vanadate concentration previously shown to enhance the activity of VSVΔ51 in these cells. Kinase inhibitor library compounds (or vehicle control) were added prior to infection with VSVΔ51 encoding firefly luciferase (VSVΔ51-FLuc). The use of VSVΔ51-FLuc allowed us to simultaneously measure the impact of treatments on virus output via a secondary assay measuring viral expression unit output from supernatants (viral expression units, akin to viral titer),[20] as well as cellular metabolic activity by resazurin assay (Alamar blue), a surrogate for detection of cell viability and cytopathic effect. Hits from this screen were defined as kinase inhibitors that counteracted vanadate's viral sensitization capacity either through a reduction in virus output or through a reduction in the cytopathic effect, or both. Kinase inhibitors that simultaneously prevented vanadate-mediated oncolysis to a threshold of 60% and decreased viral output below a log fold-change threshold of 1.5 are shown in the upper left quadrant of Figure 1B. Addition of selected kinase inhibitor hits within the upper left quadrant restored cell viability and/or fold-change viral output to levels similar to mock-treated, infected controls (Figures S1 and S2). A total of 23 small molecule kinase inhibitors were found to antagonize vanadate's viral sensitizing effect (listed in Table 1). Nearly half of the selected inhibitors (43%) target the epidermal growth factor receptor (EGFR), while several others (13%) target the downstream mitogen-activated protein kinase kinase (MAP2K or MEK1/2) (Figure 1C).

Inhibition of the EGFR pathway abrogates vanadate-mediated viral infectivity

EGFR is a receptor tyrosine kinase found on the cellular membrane, responsible for regulation of cell proliferation and survival events.[21] Given that over half of the identified hits targeted the EGFR-MEK1/2 signaling axis, we sought to first examine the activation status of these two kinases upon combined treatment with vanadate and VSVΔ51 in 786-0 cells. Densitometry following western blotting uncovered that the phosphorylation ratios of EGFR and the downstream extracellular signal-regulated kinase 1/2 (ERK1/2) were increased with vanadate and VSVΔ51 combinational treatment compared with all other conditions (Figures 1D and S3).

By western blot, we found that a concentration as low as 1 μM of the EGFR inhibitor gefitinib in human 786-0 cells was sufficient to abrogate EGFR phosphorylation induced by vanadate alone and in combination with VSVΔ51 (Figure 2A). Similar results were obtained using the ERK1/2 inhibitor UO126 albeit at higher concentrations (Figure S4). EGFR inhibition using gefitinib and erlotinib, or of MEK1/2 (using UO126), dose-dependently decreased the vanadate-enhanced growth of VSVΔ51 tagged with a GFP marker in 786-0 cells as determined by phase and fluorescent microscopy images captured 24 h post infection (hpi) (Figures 2B, S5, and S6). As expected, control

Table 1. Full list of hits of the kinase inhibitor high-throughput screen

ID	Drug	Target(s)	Developmental stage
KI1C5	Gefitinib, Iressa, ZD1839	EGFR (ERBB1, HER1), ErbB2 (TKR1, HER2, NEU)	Approved: metastatic non-small cell lung cancer
KI1C6	Lapatinib ditosylate, Tykerb, GW572016	EGFR (ERBB1, HER1), ErbB2 (TKR1, HER2, NEU)	Approved: advanced or metastatic breast cancer
KI1C10	Vandetanib, Zactima, ZD6474, AZD-6474	EGFR (ERBB1, HER1), KDR (VEGFR2, VEGFR, FLK1), RET, ABL1 (ABL), KIT (c-KIT), FLT1 (VEGFR1), FLT4 (VEGFR3), TRKA (TRK)	Approved: local or metastatic medullary thyroid cancer
KI1E2	Erlotinib HCl, CP-358774, OSI-774, Tarceva, NSC-718781, RG-1415, Ro-50-8231	EGFR (ERBB1, HER1), ErbB2 (TKR1, HER2, NEU)	Approved: metastatic non-small cell lung cancer, metastatic pancreatic cancer
KI1E10	GW2974	EGFR (ERBB1, HER1), ErbB2 (TKR1, HER2, NEU)	Pre-clinical
KI1F2	GW583340 dihydrochloride	EGFR (ERBB1, HER1), ErbB2 (TKR1, HER2, NEU)	Pre-clinical
KI1G10	BIBX 1382 dihydrochloride, Falnidamol	EGFR (ERBB1, HER1)	Clinical trial: adult solid tumors
KI2B6	PD-153035, AG-1517, Compound 32, SU-5271, ZM-252868, WHI-P79	EGFR (ERBB1, HER1)	Pre-clinical
KI2C2	BIBW-2992, Tovok, Afatinib	EGFR (ERBB1, HER1)	Approved: non-small cell lung cancer
KI2C5	CI-1033, Canertinib, PD-183805, SN-26606	EGFR (ERBB1, HER1), ErbB2 (TKR1, HER2, NEU) - Irreversible	Clinical trials: breast cancer
KI1B6	PD-184352, CI-1040	MAP2K1 (MEK1), MAP2K2 (MEK2), Erk2 (ERK, p38), RAF1 (c-Raf)	Clinical trials: lung, breast, pancreatic and colorectal cancers
KI2B11	AZD6244, ARRY-142886, AZD-6244, Selumetinib, ARRY-886	MAP2K1 (MEK1), MAP2K2 (MEK2), Erk2 (ERK, p38)	Clinical trials: carcinoma, non-small cell lung cancer, melanoma
KI2E2	SL327	MAP2K1 (MEK1), MAP2K2 (MEK2)	Pre-clinical
KI3G11	IMD 0354, IMD-0354	IKKb (IKK2)	Pre-clinical
KI2C6	SC-514	IKKb (IKK2)	Pre-clinical
KI1F4	IRAK-1/4 Inhibitor I	IRAK1 (IRAK)	Pre-clinical
TC2B6	NVP-AUY922, AUY922, VER-52296	Hsp90 inhibitor	Clinical trials: lymphoma, breast cancer, hematologic neoplasms
KI2B5	NU-7026, LY-293646	DNAPK (DNA-PKcs)	Pre-clinical
KI2B8	Sal003	eIF2a	Pre-clinical
KI2C8	Bosutinib, SKI-606, Bosulif	BCR, ABL1 (ABL), SRC (c-SRC), FGR (SRC2), LYN	Approved: leukemia
KI2D2	Ro-31-8220 mesylate, Bisindolylmaleimide IX	PKC	Pre-clinical
KI2E5	SU 4312, DMBI	KDR (VEGFR2, VEGFR, FLK1)	Pre-clinical
KI2F10	API-2, Triciribine, NSC154020, TCN, Tricibine, VQD-002	AKT1 (PKBa)	Clinical trials: leukemia, ovarian and breast cancer

cells treated with vanadate alone showed significant GFP signal, indicating high viral infection. To confirm that this effect was not due to increased cell toxicity elicited by the kinase inhibitors in combination with vanadate, Alamar blue assays were performed, and we observed that cell viability did not fall below 75% viability even at the highest concentrations of kinase inhibitors during vanadate co-treatment (Figure 2C), doses much higher than what was found to inhibit viral growth.

The above experiment was then repeated using vanadate-inhibitory concentration ranges of gefitinib and UO126, and infected cell supernatants were titered for viral output assessment by standard plaque assay (Figures 2D and S7). As previously reported, vanadate-treated 786-0 cells produced high viral titers compared with the mock control and addition of increasing concentrations of EGFR inhibitor in the presence of vanadate steadily decreased the viral titer, down by ~100-fold at 50 μM of gefitinib, a non-toxic dose (Figure 2C). Furthermore, expression of the VSV matrix (M) and nuclear (N) genes were significantly decreased upon addition of gefitinib compared with vanadate-only-treated cells 24 hpi as measured by qPCR (Figure 2E). In line with previous observations that vanadate potentiates other RNA-based viruses, we found that EGFR inhibition also abrogated the vanadate-mediated sensitization of 786-0 cells to measles virus (Figures S8 and S9).

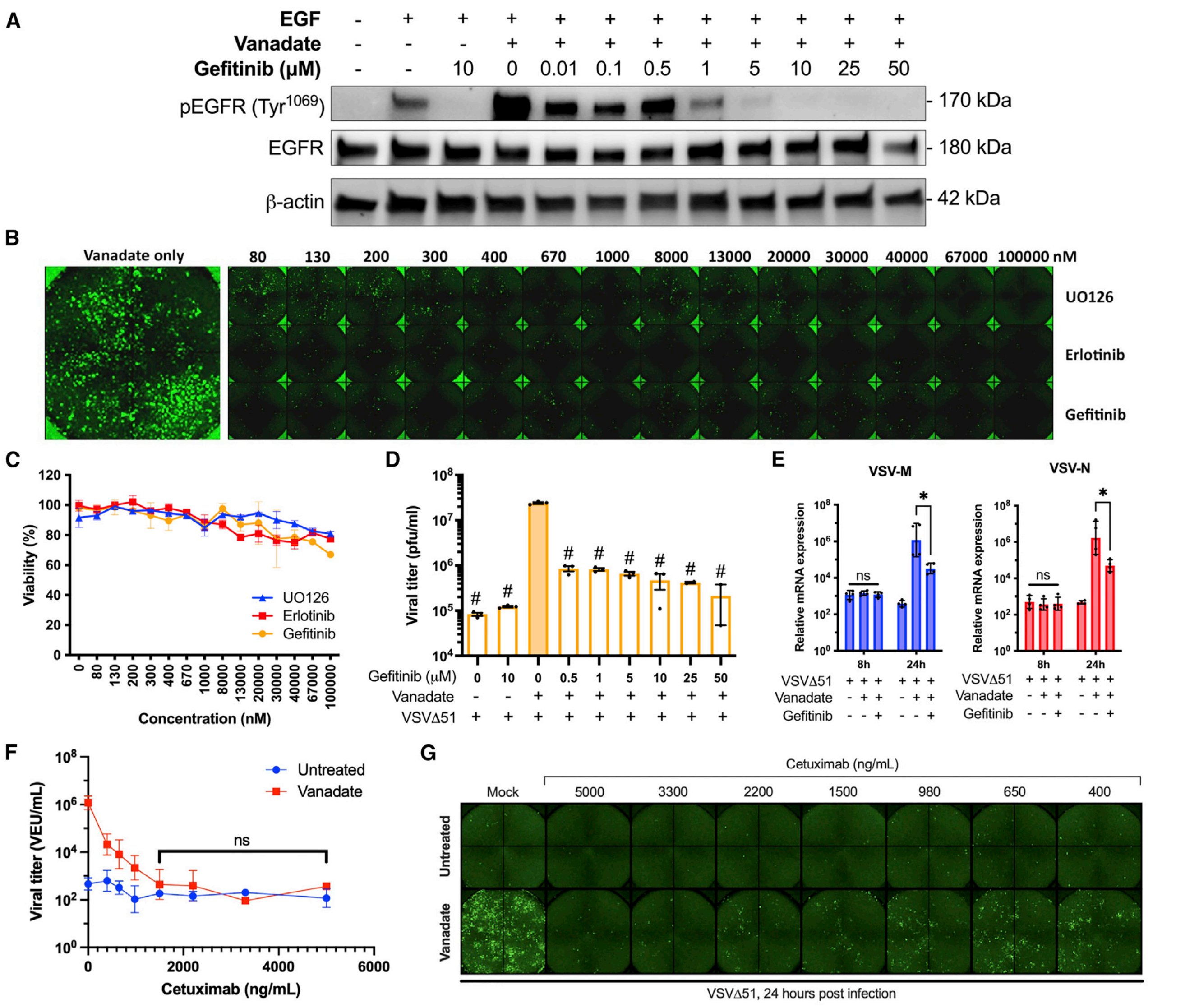

Figure 2. Inhibition of the EGFR pathway abrogates vanadate-enhanced viral infectivity

(A) Human 786-0 renal carcinoma cells were pre-treated with or without vanadate (150 μM) and varying concentrations of gefitinib (0–50 μM) as indicated for 4 h. Cells were then treated with 10 ng/mL EGF for 10 min and lysates were probed by western blot for phosphorylated EGFR, total EGFR, and β-actin. (B and C) 786-0 cells were simultaneously treated with varying concentrations of gefitinib, erlotinib or UO126 (0–100 μM) and vanadate (150 μM). Four hours later, cells were infected with VSVΔ51-GFP (MOI 0.1). (B) Fluorescence images were captured 24 hpi. (C) Cell viability was measured by resazurin (Alamar blue) assay and expressed relative to untreated, uninfected cells. (D) Supernatants were collected and titered by viral plaque assay (n = 3, mean ± SD; $^{*}p < 0.05$, $^{\#}p < 0.0001$; one-way ANOVA compared with the infected VSVΔ51 + vanadate only condition as indicated by the filled bar). (E) 786-0 cells were treated ± vanadate (150 μM) ± gefitinib (10 μM), then infected with VSVΔ51-GFP (MOI 0.1) 4 h later. RNA was extracted from cell lysates at 8 and 24 hpi and probed for VSV-M and VSV-N gene expression by qPCR (n = 4, mean ± SD; $^{*}p < 0.05$, $^{**}p < 0.01$ by one-way ANOVA, n.s. denotes no significance). (F and G) 786-0 cells were pre-treated with or without vanadate (150 μM) and varying concentrations of anti-EGFR antibody cetuximab (500–5000 ng/mL) for 4 h, then infected with VSVΔ51 (MOI 0.05). (F) Viral titer was determined 40 hpi by high-throughput titration (mean ± SD; ns = no significance by two-way ANOVA). (G) Representative fluorescence images were captured 24 hpi.

To test whether therapeutic antibodies against EGFR (cetuximab) could achieve the same effect as chemical inhibitors, 786-0 cells were pre-treated with vanadate and varying concentrations of cetuximab (50 ng/mL–5 μg/mL) for 4 h, then infected with VSVΔ51 (MOI 0.05). While cetuximab had inhibitory effects on its own at higher concentrations, the ability for vanadate to increase VSVΔ51 viral titer was greatly abrogated across all doses of cetuximab, even at a minimal concentration of 400 ng/mL (Figures 2F and 2G). Altogether, these data strongly support a key role of EGFR signaling in eliciting vanadate's viral sensitizing effects.

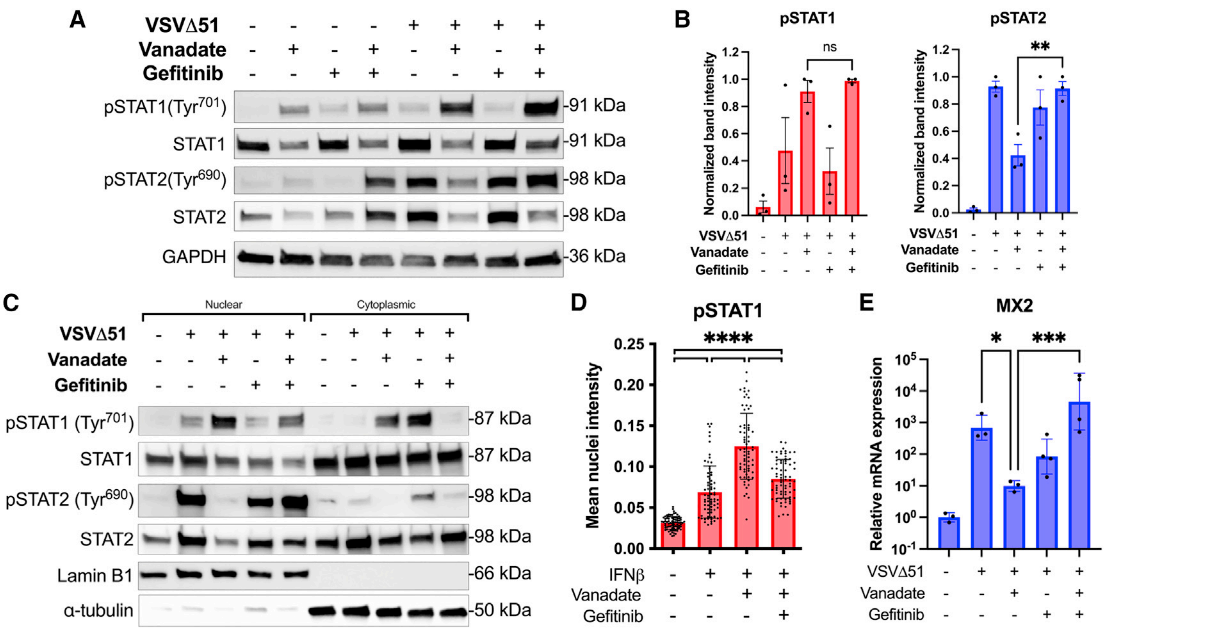

Figure 3. Vanadate regulates STAT1 and STAT2 through EGFR to modulate the interferon response

(A–C and E) Human renal carcinoma 786-0 cells were treated ± vanadate (150 μM) ± gefitinib (10 μM), then subsequently infected with or without VSVΔ51-GFP (MOI 0.01). (A) Total cell lysates were collected 24 hpi and probed for phosphorylated STAT1, total STAT1, phosphorylated STAT2, total STAT2, and GAPDH by western blot. Representative blots are shown. (B) Bands were quantified by densitometry relative to the actin-loading control and expressed as a phosphorylated: total STAT1/2 ratio (n = 3, mean ± SD; ns = no significance, $^{**}p < 0.01$ by one-way ANOVA). (C) Lysates were fractionated for nuclear and cytoplasmic fractions and probed for phosphorylated STAT1, total STAT1, phosphorylated STAT2, total STAT2, lamin B1, and alpha-tubulin by western blot. (D) 786-0 cells were treated ± vanadate (300 μM) ± gefitinib (20 μM) for 4 h, then with IFNβ (100 U/mL) for 1 h. Cells were fixed and stained for phosphorylated STAT1 and nuclei with DAPI by immunofluorescence. Phospho-STAT1 nuclear intensity was quantified, and mean nucleus intensity graphed (n = 3, mean ± SD; $^{****}p < 0.0001$ by one-way ANOVA). Representative images can be found in Figure S11. (E) RNA was extracted from cell lysates 24 hpi and probed for *MX2* mRNA expression by qPCR (n = 3, mean ± SD; $^{***}p < 0.001$ by one-way ANOVA).

Vanadate regulates STAT1 and STAT2 through EGFR to modulate the IFN response

The IFN-1 response is dependent on the phosphorylation and subsequent formation of STAT1-STA2 heterodimers. When combined with IFN response factor 9 (IRF9), the formed interferon-stimulated growth factor 3 (ISGF3) complex then translocates to the nucleus where it regulates the transcription of IFN-stimulated genes, including the IFN-induced GTP-binding protein MX2. Conversely, the IFN-2 response, responsible for induction of proinflammatory genes such as CXCL9 and CXCL10 chemokines, is propagated through phosphorylated STAT1 homodimers binding to the respective IFN gamma-activated sequences (GAS).[22] In a previous study, we found that vanadate leads to increases in STAT1 phosphorylation with concomitant decreases in STAT2 phosphorylation levels. This correlated with a shift from IFN-1 type toward IFN-2 type virus-induced gene expression profiles in a variety of cell types.[15] Evidence suggest that activation of the EGFR pathway and its downstream players including ERK1/2 compromise the antiviral IFN-1 defense through regulation of STAT signaling.[23,24] Therefore, we next sought to assess STAT dynamics in response to EGFR inhibition.

Human 786-0 cells pre-treated with or without vanadate and/or gefitinib were infected with VSVΔ51 and protein extraction was performed 24 hpi. As found in previous studies,[15] probing whole-cell lysates by western blot showed that following VSVΔ51 infection, vanadate increases phosphorylation of STAT1 and decreases phosphorylation of STAT2. While EGFR inhibition by gefitinib did not impact STAT1, it was interestingly able to rescue the otherwise decreased levels of STAT2 phosphorylation caused by vanadate/virus infection (Figures 3A, 3B, and S10). We were then interested to see whether EGFR inhibition could reduce vanadate/virus-induced STAT1 nuclear translocation.[15] Nuclear/cytoplasmic fractionation in samples treated with the same regimen indeed revealed increased phosphorylated and total STAT1 nuclear localization upon vanadate/virus treatment, which was abrogated by EGFR blockade by gefitinib (Figure 3C). In parallel, normalization of STAT2 nuclear translocation in accordance with rescued STAT2 phosphorylation by gefitinib treatment was also observed. This was further confirmed using immunofluorescence. We found that upon stimulation using IFNβ, vanadate treatment led to concentrated nuclear localization of phosphorylated STAT1 compared with cells only treated with IFNβ, where nuclear phospho-STAT1 signal was more diffuse (Figures 3D and S11). However, upon addition of gefitinib, the concentration of nuclear phosphorylated STAT1 decreased markedly. Finally, coinciding with these observations, the addition of gefitinib restored the virus-induced upregulation of the downstream IFN-1

gene *MX2,* which is otherwise suppressed by vanadate in response to VSVΔ51 infection, as determined by qPCR (Figure 3E). Altogether, these data support the possibility that EGFR signaling induced by vanadate/virus treatment leads to immunomodulation and improved viral growth by altering the phosphorylation status of STAT1/STAT2.

Vanadate promotes proinflammatory cytokine production through EGFR-NFκB activation

To gain additional insight into the vanadate viral sensitizing mechanism, we performed *in silico* analysis on a previously published microarray dataset looking at the impact of combined vanadate and VSVΔ51 treatment in 786-0 cells.[15] We selected genes that increased or decreased at least 3-fold in abundance in cells receiving combined vanadate and VSVΔ51 treatment. Then using the published TFactS tool,[25] we found that the top predicted transcription factor to correlate with this differential gene expression profile was STAT1, confirming our previous and current findings. However, our *in silico* analysis also identified nuclear factor (NF)-κB as the second most likely involved transcription factor (Figures 4A, S12, S13, and S14). This was interesting given that two kinase inhibitor hits from our reverse screen (Table 1) target IκB kinase beta (IKKβ). Like gefitinib and UO126, the two IKKβ inhibitors, IMD-0354 and SC-514, reduced VSVΔ51-GFP infectivity as measured by GFP expression, even in the presence of vanadate (Figures 4B and S15). This effect occurred within a broad drug treatment range leading to no more than a 25% decrease in cell viability (Figure 4C).

We consequently sought to look at the impact of vanadate and treatments antagonizing EGFR on NF-κB nuclear translocation. Indeed, probing for NF-κB in nuclear and cytoplasmic fractions by western blot demonstrated that vanadate increases the quantity of NF-κB available in the nucleus, an effect that was abrogated with gefitinib treatment (Figure 4D). The inhibitor of NF-κB alpha (IκB-α) is a cytosolic inhibitor protein that regulates NF-κB signaling by masking its nuclear localization signal and its subsequent nuclear translocation.[26,27] Interestingly, EGFR phosphorylation has also been established to increase NF-κB signaling through the degradation of IκB-α.[28,29] To explore this linkage in the context of vanadate's viral sensitizing mechanism, we probed whole-cell lysates for IκB-α by western blot. Combinatorial treatment of vanadate and VSVΔ51 led to a significant decrease in IκB-α protein levels, yet when EGFR was inhibited using gefitinib, cellular levels of IκB-α were restored (Figures 4E and S16).

Our previous study established that the combined treatment of vanadate with VSVΔ51 upregulates proinflammatory cytokines such as IFNβ several hours after the infection event.[15] These proinflammatory cytokines are in part stimulated through the NF-κB axis, which is itself downstream of EGFR. We therefore tested to see whether EGFR inhibition was able to abrogate the expression of NF-κB target genes. Quantification by qPCR revealed that the addition of gefitinib negated increases in proinflammatory IFNβ, IL-1b, IL-6, TNF-α and CCL5 mRNA levels induced by vanadate (Figure 4F), all of which are in part stimulated by NF-κB.[30] Moreover, one of the most striking phenotypes of tumor cells treated with vanadate in combination with VSVΔ51 is an increase in the transcription of T cell chemoattractant chemokines, namely the IFN-2-induced chemokines CXCL9 and CXCL10.[15] Guided by evidence that STAT1 and NF-κB can synergize to upregulate both genes,[31,32] we explored whether vanadate also used this mechanism to increase these chemokines through EGFR. Transcriptional expression of genes encoding immune cell-attracting cytokines CXCL9 and CXCL10 were also assessed by qPCR. Indeed, gefitinib negated the increased transcription of CXCL9 and CXCL10 induced by vanadate (Figure 4F). When secreted CXCL10 was measured by ELISA in the supernatant, the same trend was reflected (Figure 4G). Together, these results provide evidence to support that vanadate facilitates increased NF-κB signaling favoring a proinflammatory profile in treated tumor cells by regulating IκB-α through EGFR activation.

Gefitinib reduces vanadate's effects on VSVΔ51 spread *in vivo*

Given gefitinib's ability to abrogate sensitization to VSVΔ51 by vanadate *in vitro*, we sought to investigate whether gefitinib could block the effects of vanadate-enhanced VSVΔ51 oncolytic virotherapy in more physiologically relevant contexts. Upon confirming that the addition of gefitinib was able to reduce vanadate viral sensitization in colon CT26WT carcinoma cells when administered pre- and post-infection *in vitro* (Figures S17 and S18), we found that CT26WT colon mouse tumor cores pre-treated with vanadate and treated with gefitinib 4 h prior to infection were also capable of curbing vanadate-mediated VSVΔ51 viral spread *ex vivo* (Figures 5A and 5B). This effect was also achieved with UO126 treatment in CT26WT tumors (Figure S19).

To investigate whether gefitinib could also abrogate the enhancing effect of vanadium on VSVΔ51 spread *in vivo*, 8-week-old BALB/c mice were implanted subcutaneously with CT26WT tumors and underwent a similar treatment regimen consisting of vanadyl sulfate and VSVΔ51-FLuc administered intratumorally, along with gefitinib (100 mg/kg) 4 h prior to infection. At 24 hpi, infection was assessed using a live *in vivo* imaging system (IVIS). We did not investigate tumor progression to avoid potential confounding anti-tumor effects of gefitinib treatment. Consistent with our previous findings, mice receiving combinational treatment and gefitinib after infection significantly lowered viral-associated luciferase signal compared with mice not receiving any gefitinib (Figures 5C and 5D). Together, both our *ex vivo* and *in vivo* data support that gefitinib can reverse the effects of vanadate in combination with VSV in animal models.

DISCUSSION

Identification of EGFR signaling to vanadate viral sensitization

Despite the exciting multimodal therapeutic effects of OVs for cancer therapy, their success has yet to be fully realized due to variable response rates. To potentiate their anti-tumor effects, our group and others have previously explored the use of approved or novel compounds that can lead to the selective and transient knockdown of antiviral defenses to allow OVs to gain a foothold within the tumor.[12] Vanadium-based compounds such as vanadate or vanadyl sulfate have shown promise for combinational therapy with RNA-based OVs in particular, such as VSV and Newcastle disease virus

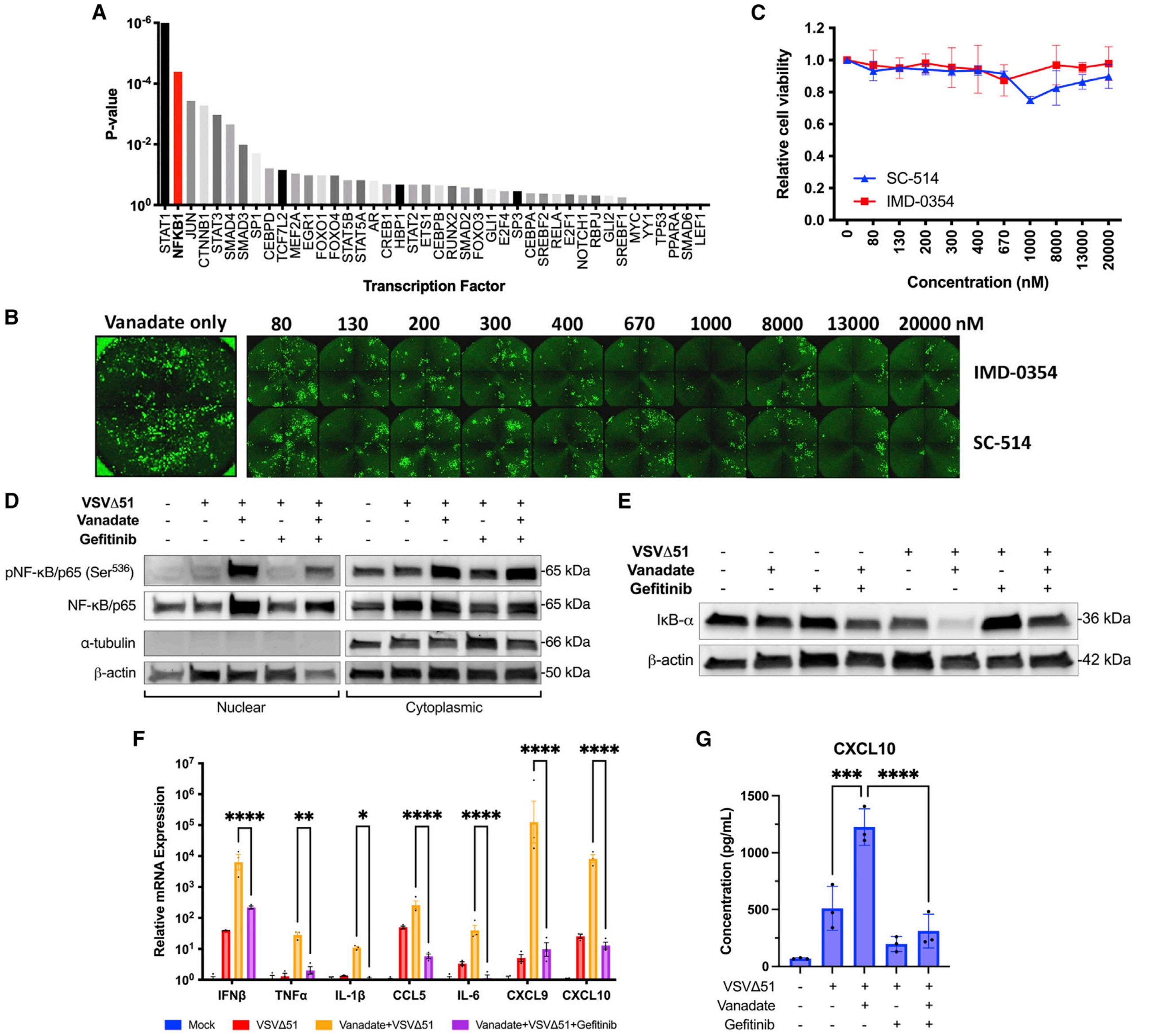

Figure 4. Vanadate promotes proinflammatory cytokine production through EGFR-NFκB activation

(A) Previously acquired microarray data published by Selman et al.[15] was analyzed using a computational script that selected genes that were of increased or decreased abundance 3-fold upon vanadate treatment from the infected only control. Gene lists were input into TFactS.[25] The p value for each analyzed transcription factor was plotted. (B, C) 786-0 cells were simultaneously treated with varying concentrations of IMD-0354 or SC-514 (0–2000 nM) and vanadate (150 μM) (n = 2, mean ± SEM). Four hours later, cells were infected with VSVΔ51-GFP (MOI 0.1). (B) Fluorescence images were captured 24 hpi. (C) Cell viability was measured by resazurin (Alamar blue) assay and expressed relative to untreated, uninfected cells. (D–F) 786-0 cells were treated ± vanadate (150μM) ± gefitinib (10μM). Four hours later, cells were infected with VSVΔ51-GFP (MOI 0.1). (D) Cells were lysed 24 hpi, fractionated for cytoplasmic and nuclear fractions, then probed for phosphorylated NF-κB/p65, NF-κB/p65, lamin B1, and α-tubulin. (E) Whole-cell lysates extracted 24 hpi and probed for IκB-α and β-actin. (F) Cells were lysed at 24 hpi and analyzed for mRNA expression of NF-κB related and proinflammatory genes by quantitative polymerase chain reaction (n = 3, mean ± SD; **p < 0.01, ***p < 0.001, ****p < 0.0001 by one-way ANOVA).
(G) Supernatant was collected 24 hpi and CXCL10 concentration was assessed by ELISA (n = 3, mean ± SD; ****p < 0.0001 by one-way ANOVA).

(NDV).[15,33] While we have previously shown that vanadium compounds elicit their OV-enhancing effects via a shift in IFN-1 toward IFN-2 transcriptional profiles by altering STAT1/2 expression and phosphorylation status, the specific mechanistic cascade leading to this effect remained unclear. In this study, we used a systems biology approach to successfully identify the EGFR pathway and its downstream signaling cascade as a crucial player in the viral sensitizing effect of vanadium compounds. This previously unappreciated mechanistic insight adds to our understanding of key signaling pathways that contribute to successful OV therapy regimens and will

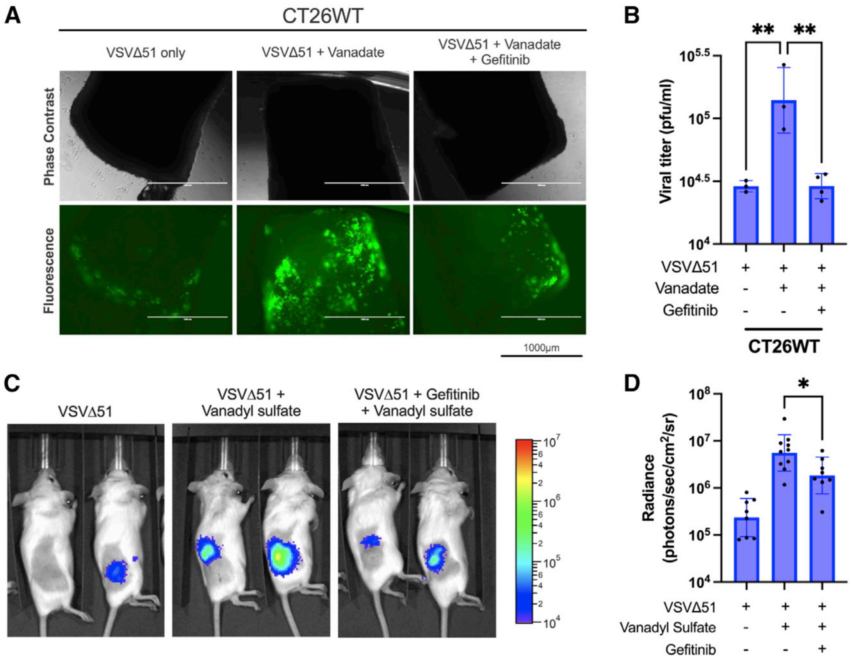

Figure 5. Gefitinib reduces the effects of vanadium-VSVΔ51 combination therapy *in vivo*

(A and B) CT26WT tumor cores were obtained from implanted Balb/c mice and treated *ex vivo* with vanadate (300 μM) ± gefitinib (50 μM). Cores were infected 4 h later with 3e4 plaque forming units (pfu) of VSVΔ51-GFP per core. (A) Fluorescence images were taken 24 hpi; scale bar, 1000 μm. (B) Supernatant was collected 48 hpi and viral titer was assessed by plaque assay (n = 3, mean ± SEM; $^{**}p < 0.01$ by one-way ANOVA). (C and D) Balb/c mice were implanted subcutaneously with CT26WT and allowed to progress to 100 mm^3. Mice were then injected intratumorally with vanadyl sulfate (50 mg/kg) ± gefitinib (100 mg/kg) for 4 h. Mice were then injected intratumorally with VSVΔ51-FLuc (1e8 pfu/tumor). At 24 hpi, mice were imaged using a live imaging system (IVIS) for luminescence activity. (C) Absolute luminescence was log-transformed and graphed (n = 8–10, mean ± SEM; $^{*}p < 0.05$ by one-way ANOVA). (D) Representative luminescence images are shown.

facilitate pre-clinical and clinical development of vanadium compounds as enhancers of OV therapy. Our screen also identified eight additional targets outside of EGFR that abrogated vanadium viral sensitizing activity (Table 1), some of which can play a role in viral lifecycle and antiviral pathway regulation (e.g., eIF2a, AKT1) and provide avenues for further investigation.[34]

In addition to the insulin-mimetic effects of vanadium that have been well explored in clinical trials,[35] investigations have also suggested direct anti-cancer effects of vanadium compounds through *in vitro* and *in vivo* experimentation.[19,36–39] However, their potential use in immuno-virotherapy has only recently been uncovered.[15,33] Vanadium-based compounds are potent competitive PTP inhibitors that naturally impact classes of receptor tyrosine kinases, including the ErbB family. Vanadate has been previously established to sustain EGFR activation and its downstream signaling (Figure 1D).[40,41]

Guided by the mounting evidence supporting the role of the EGFR axis in oncogenesis and metastasis, several small molecule EGFR modulators have been developed and clinically approved in the past few decades.[42] Gefitinib, lapatinib, vandetanib, erlotinib, and afatinib are among the lead compounds identified in our screen and are clinically approved for the treatment of a variety of malignancies, particularly in tumors found to have EGFR overexpression. While several groups have explored the targeting of oncolytic viruses to take advantage of EGFR upregulation in cancer for increased therapeutic specificity,[43–45] EGFR inhibitors have not typically been explored to modulate oncolytic virotherapy efficacy. Our study draws a link between virus-induced EGFR activation, increased oncolytic virus activity, and the ability to "tune" oncolytic virus activity by pharmacologically controlling the EGFR pathway.

In support of our proposed mechanism of vanadate-mediated OV enhancement via the EGFR pathway, there is ample evidence demonstrating that activation of the EGFR pathway and its downstream effectors sidetracks the antiviral system and promotes viral propagation. For instance, several viruses activate EGFR as part of their viral lifecycle to allow for their propagation.[46–49] Poxviruses, which are currently used in oncolytic virotherapy, in particular encode vaccinia growth factor, which has been shown to activate EGFR.[50] In a study by Wang et al., tyrosine phosphorylation of the key IFN effector STING (STimulator of INterferon Genes) by EGFR was required for its proper cellular trafficking and downstream signaling.[51] Without this phosphorylation by activated EGFR, STING was alternatively trafficked to autophagosomes, and cells were unable to mount a proper antiviral response against HSV infection. By the same token, inhibition of EGFR by genetic or pharmacological means has also been reported to activate the interferon pathway in several contexts.[23,52,53] While sustained virus-induced EGFR signaling is likely in part required for its OV-enhancing effect, it is likely that vanadate requires the modulation of other signaling components. Investigation into the other specific phosphatases that vanadate may inhibit to confer viral sensitization effects is currently under way for the development and refinement of this therapeutic strategy.

Analyzing the immunomodulatory mechanism of vanadate via EGFR activation

From our findings, we elucidate three molecular events through which vanadate sensitizes cells to oncolytic virotherapy via EGFR activation: 1) dephosphorylation of STAT2, 2) induction of STAT1 nuclear accumulation, and 3) degradation of IκB-α to increase nuclear NF-κB signaling (see graphical abstract). During viral infection, IFN-1 cytokine binding to its respective IFN receptor (IFNAR1/2) triggers STAT1 and STAT2 phosphorylation and heterodimerization. We demonstrate that through EGFR activation, vanadate inhibits

STAT2 phosphorylation to impair IFN-1 signal transduction. Without the phosphorylation of STAT2, the activity of the ISGF3 complex and subsequent antiviral defenses are abolished.[54–56] Accordingly, EGFR blockade restores STAT2 phosphorylation and subsequent transcription of IFN-1 genes (Figures 3A, 3B, and 3E). As there is little known about the direct EGFR-STAT2 relationship, it is likely that an intermediate effector exists. For example, hyperactivation of the Ras/MEK pathway downstream to EGFR has been suggested to inhibit STAT2 transcriptional availability,[57] and contribute to VSVΔ51 oncolysis.[58] Further investigation into this relationship would broaden our understanding of STAT2 regulation of the IFN-1 responses.

Our computational analysis on microarray data identified STAT1 as the most likely involved transcriptional factor in the vanadate viral sensitizing mechanism, in accordance with this and previous studies (Figures 4A and S13). Several groups have already shown that activation of the EGFR cascade culminates in increased STAT1 activation and expression.[59–61] This is particularly relevant in the context of vanadate-based oncolytic virotherapy, since we have previously shown that vanadate's mechanism of action involves the increased phosphorylation and nuclear localization of STAT1 upon VSVΔ51 infection, both of which were also observed in this study (Figure 3).[15] In accordance with these findings, identified phosphatases targeting STAT1, such as Src homolog 2 domain-containing phosphatase 2 (SHP-2),[62] have been linked with impaired antiviral defenses through EGFR activation.[63,64]

At first glance, increased NF-κB signaling by vanadate appears to be counterintuitive for oncolytic potentiation as other established viral sensitizing compounds such as dimethyl fumarate (DMF) operate by inhibiting NF-κB nuclear translocation.[65,66] However, our results are in agreement with other studies that the JAK/STAT pathway is the key, dominant mechanism in controlling the IFN-1 response.[67,68] In fact, retaining NF-κB nuclear availability allows vanadate to directly upregulate the secretion of proinflammatory cytokines to ultimately increase immune cell infiltration to the tumor site, a unique feature of vanadium compounds.[15] Through the cooperative signaling of increased STAT1 (Figure 3D) and NF-κB (Figure 4D) nuclear availability, vanadate achieves synergistic induction of proinflammatory genes controlled by GAS or NF-κB promoters.[69] In the case of two IFN-2 cytokines, CXCL9 and CXCL10, the interaction between STAT1 and NF-κB has shown to increase recruitment of a third protein, CREB-binding protein to subsequently increase RNA polymerase II transcription activity at their respective promoters.[31,32] Indeed, we were able to demonstrate that EGFR blockade, presumably through inhibiting vanadate's STAT1 and NF-κB nuclear accumulation, significantly reduced IFN-2 cytokine transcription and secretion (Figures 4F and 4G).

Implications for the advancement of vanadium-based oncolytic virotherapy

Our findings introduce previously unappreciated considerations and opportunities for the advancement oncolytic virotherapy regimens. By establishing EGFR as an integral regulator of oncolytic VSV growth, it is possible that EGFR status could influence patient response to treatment regimens employing VSV and potentially other RNA-based OVs like measles and NDV. EGFR is frequently mutated in cancer, its hyperactivation confers a survival benefit to cancer cells and has been established as a resistance marker to standard cancer therapy.[70–74] Interestingly, activation of EGFR has been shown to be necessary for the replication of several OVs and exploited to improve OV selectivity toward cancer cells. Alternate activation of the downstream effector ERK1/2 has also been shown to sensitize endothelial cells to OV therapy.[75] Taken together with our findings, this further suggests the possibility that patients harboring mutations with hyperactivated EGFR may naturally be more susceptible to VSVΔ51 oncolytic virotherapy, a hypothesis that warrants further clinical investigation. Conversely, our results would then also naturally suggest that patients who are undergoing treatment with an EGFR inhibitor (e.g., gefitinib, erlotinib, cetuximab) are less likely to respond to vanadium/VSVΔ51 combination therapy. Furthermore, we consider that this aspect could potentially be exploited to control immune adverse events during oncolytic VSV clinical trials. EGFR inhibitors are advantageous candidates in that they are already clinically approved and additionally offer inherent anti-cancer properties; however, whether they offer better counter-therapy than other antivirals, remains to be determined.

MATERIAL AND METHODS

Drugs and chemical reagents

The vanadium-based compound used in this study was sodium orthovanadate (Na_3VO_4, Sigma-Aldrich, cat. 450243) dissolved in water and pH adjusted to 10 at 150 μM. For *in vivo* studies, vanadium sulfate at 50 mg/kg ($VOSO_4$, Sigma-Aldrich, cat. 204862) was used. The remainder of drugs, chemicals, and cytokines used are listed in Key Resources Table (Table S1).

Cell lines

The 786-0 (human male renal cell adenocarcinoma, cat. CRL-1932), CT26WT (murine colon carcinoma, cat. CRL-2638), and Vero (African green monkey kidney cells, cat. CCL-81) were acquired from the American Type Culture Collection. These cells used DMEM (HyClone cat.10-013) supplemented with 1% (v/v) penicillin-streptomycin (Gibco), 30 mM HEPES buffer, and 10% (v/v) serum composed of 3 parts HyClone newborn calf serum (Thermo Fisher, cat. SH3011803) and 1 part fetal bovine serum (Gibco, cat. 12483020). Cell lines were maintained in 37°C and 5% CO_2 conditions in a humidified incubator.

Oncolytic virus

The Indiana serotype of VSV harboring a deletion of methionine 51 in the M protein (VSVΔ51) and insertion of GFP or FLuc were used throughout this study.[76] All viruses were propagated on Vero cells and purified on 5%–50% Optiprep (Sigma-Aldrich, St. Louis, MO) gradients. All viral titers were determined by standard plaque assay according to published protocol.[77] Measles virus of the Schwartz strain expressing GFP was obtained as a generous gift from Dr.

Guy Ungerechts of the Ottawa Hospital Research Institute (Ottawa, Canada).

Reverse screen strategy using kinase inhibitor library

The Ontario Institute for Cancer Research small molecule library was generously supplied by Rima Alawar via Dr. William Stanford of the Ottawa Hospital Research Institute. The library is composed of 480 kinase inhibitors and 320 tool compounds from various developmental stages (e.g., approved, pre-clinical, clinical). We plated 3×10^4 786-0 cells per well of a 96-well microplate and allowed them to adhere overnight. Cells were pre-treated with screen compounds (1 μM) and vanadate (125 μM). Four hours later, cells were infected with VSVΔ51-FLuc at an MOI of 0.1. Infectivity was quantified by high-throughput titration (see below) and cell viability using resazurin (Millipore Sigma, cat. SI03200) was measured 48 hpi.[20] The experiment was conducted in triplicate. Every microplate included a series of positive (vanadate + VSVΔ51) and negative (untreated) controls, with a total of 80 library compounds.

High-throughput titration

Using opaque white bottom 96-well microplates (Thermo Fisher, cat. 07-200-628), Vero cells were seeded at a density of 2.5×10^4 cells/well; 20 μL of the supernatant from the sample of interest was transferred onto the Vero cells and incubated for 5 h at 37°C and 5% CO_2. An amount of 25 μL luciferin solution (2 mg/mL constituted in sterile PBS, Cedarlane Labs, cat. 122799(PE)) was subsequently added and mean luminescence was analyzed 30 s later. Results were normalized against a standard curve of samples with known titers and normalized to an uninfected, untreated well. Refer to published protocol for further details.[20]

In silico analysis

Microarray data from a previous study was processed.[15] A list of genes of increased or decreased abundance more than 3-fold between the VSVΔ51-infected condition and the vanadate and VSVΔ51-combinational condition was generated, then input into the TFactS computational database script to generate statistical values for each transcription factor. Related gene lists for each respective transcription factor were obtained from the published TFactS catalogue. Heatmaps of relative gene expression were generated using the R-studio pheatmap package. Refer to original published study for further details.[25]

Cell viability assay

Metabolic activity of cells was measured 48 hpi by adding 1:10 dilution resazurin metabolic dye (Millipore Sigma, cat. SI03200) to treated cells and incubated for 2 h. Fluorescence was measured at 590 nm upon excitation at 530 nm using a BioTek Microplate Reader and Gen5 2.07 software (Norgen BioTek Corp, ON, Canada). Background signal was adjusted for by subtracting readings from wells containing only media.

Quantitative real-time polymerase chain reaction

RNA was extracted from samples using the QIAGEN RNeasy kit (Qiagen, cat. 74106) according to the manufacturer's protocol and quantified using a Nanodrop ND-1000 spectrophotometer (Thermo Fisher Scientific, Rockford, IL). Corresponding cDNA was generated using the RevertAid H Minus First Strand cDNA Synthesis Kit (Thermo Fisher, cat. K1632). Real-time PCR reactions were performed using respective primers (Table S2), Applied Biosystems PowerUp SYBR Green Master Mix (Thermo Fisher, cat. A25776) in a 7500 Fast Real-Time PCR system (Applied Biosystems, Foster City, CA). Relative gene expression was normalized to *GAPDH*, and fold-induction calculated relative to an uninfected and untreated condition.

Enzyme-linked immunosorbent assay

The 786-0 cells were treated with respective drugs for 4 h and infected with VSVΔ51. After 24 h, supernatants of cells were collected and analyzed for CXCL10 concentration using the Human CXCL10/IP-10 DuoSet Assay kit (R&D Systems, cat. DY266). The assay was performed according to the manufacturer's protocol with a 1:30 dilution. Absorbance was read using the Multiskan Ascent Microplate Reader (MXT Lab Systems) at 450 nm and corrected for plate imperfections at 540 nm.

Immunoblotting and immunoprecipitation

Samples were washed twice with cold PBS and lysed for 10 min at 4°C using 50 mM HEPES, 150 mM NaCl, 10 mM EDTA, 10 mM Na4P2O7, 100 mM NaF, 2 mM Na3VO4, protease inhibitor cocktail (Roche), phosphatase inhibitor cocktail (Cell Signaling Technology, cat. 5870 S), and 1% Triton X-100. Cells were scraped and the collected lysate was centrifuged. For nuclear and cytoplasmic fractionation, the NE-PER Nuclear and Cytoplasmic Extraction Kit (Thermo Fisher Scientific) was used according to the manufacturer's protocol. Supernatant was collected and the protein quantified using the Pierce BCA Protein Assay Kit (Thermo Fisher, cat. 23225); 20 μg was loaded with 4X NuPAGE LDS Sample Buffer (Thermo Fisher, cat. NP0007) into 4% to 15% Mini-PROTEAN Gels (Bio-Rad, Mississauga, ON), electrophoresed using the Mini Trans-Blot Cell system (Bio-Rad, Mississauga, ON), and transferred onto nitrocellulose membrane using the Trans-Blot Turbo RTA Mini Transfer Kit according to the manufacturer's protocol (Bio-Rad, cat. 1704270). Blots were subsequently blocked with 5% BSA and probed with respective primary and secondary antibodies as listed in the Key Resources Table (Table S1). Bands were visualized using Clarity Western ECL Substrate (Bio-Rad, cat. 1705061) on a ChemiDoc Touch Imaging System (Bio-Rad, Mississauga, ON).

Immunocytochemistry

Cells were seeded on 12-mm glass round coverslips (Thomas Scientific, cat. 64-0712), then treated with specified reagents. After washing twice with PBS* (PBS with 1 mM $CaCl_2$ and 500 μM $MgCl_2$), cells were fixed using 4% paraformaldehyde for 30 min, permeabilized using a 0.2% Triton-X 100 in 200 mM glycine/PBS* solution for 8 min, then quenched in 200 mM glycine/PBS*. Samples were then blocked using 5% BSA/PBS* for 1 h at room temperature, then incubated with respective primary antibody overnight in a humidified chamber at 4°C as listed in the Key Resources Table (Table S1). Corresponding

secondary antibodies were applied for 1 h, then samples were mounted onto glass slides and counterstained using Prolong gold anti-fade with DAPI (Molecular Probes). Slides were imaged using Zeiss Axiocam HRM Inverted fluorescent microscope (Zeiss, Toronto, Canada) and Axiovision 4.0 software. Images were processed using ImageJ. Nuclear: cytoplasmic signal quantification processes were performed using CellProfiler (Massachusetts Institute of Technology, Cambridge, USA).

Ex vivo tumor model

Eight-week-old healthy, female Balb/c mice were implanted with subcutaneous CT26WT cells. Mice were sacrificed after reaching a tumor volume of 1000 mm^3. Tumor tissues were extracted from the mice, cut into 2-mm slices and 2 mm $\times$ 2 mm cores were taken using a punch biopsy tool. Cores were maintained in humidified incubators at 37°C, 5% CO_2 in DMEM supplemented with 10% serum, 30 mM HEPES, 1% (v/v) penicillin-streptomycin, and 0.25 mg/L amphotericin B. Cores were treated with their respective drugs at indicated timepoints, then infected with VSVΔ51-GFP (3e4 pfu/core for CT26WT). Fluorescence images were taken 24 hpi and supernatant stored at −80°C 48 hpi for viral titer plaque assay.

In vivo tumor models

Female 8-week-old Balb/c mice (Charles River Laboratories) were implanted subcutaneously with CT26WT cells and tumors allowed to progress to 100 mm^3, about 11 days. Tumors were treated intratumorally with vanadyl sulfate (50 mg/kg) and/or gefitinib (100 mg/kg) at various time points. Tumors were then injected with VSVΔ51-FLuc (1e8 pfu/tumor) intratumorally. After 24 h, mice were anesthetized and imaged using a live *in vivo* imaging system (Perkin Elmer). Bioluminescent signal intensity was quantified and analyzed using Living Image v2.50.1 software. All experiments were performed in accordance with the University of Ottawa Animal Care and Veterinary Service guidelines for animal care under the protocols OHRI-2264 and ORI-2265.

Quantification and statistical analysis

Statistical analyses were performed using Prism 8 (GraphPad, San Diego, CA) software. Experiments involving viral titer, absolute luminescence, and relative mRNA expression were log-transformed before statistical tests were performed as indicated by the figure legends, including Student's t test, and one-way and two-way ANOVA, according to experimental conditions. Error bars represent the SEM unless otherwise noted. A p value less than 0.05 was considered statistically significant.

Data and materials availability

The lead contact for this project who will be responsible for distribution of materials, datasets, and protocols used in the manuscript is Boaz Wong (boaz.wong@uottawa.ca). This study did not generate new unique reagents. All raw data, including uncropped western blot images and data values, have been deposited at Mendeley Data and are publicly available as of the date of publication (https://doi.org/10.17632/zzpvtnmdw7.1). This paper does not report original code. The microarray data that were analyzed have previously been deposited in the NCBI-Gene Expression Omnibus database (GEO: GSE97327). Any additional information required to reanalyze the data reported in this paper is available from the lead contact upon request.

SUPPLEMENTAL INFORMATION

Supplemental information can be found online at https://doi.org/10.1016/j.omto.2022.04.004.

ACKNOWLEDGMENTS

The graphical abstract schematic was created using BioRender.com (Toronto, ON, Canada) licensed to B.W. (agreement # QL23OS0 PG3). The authors thank Michael Phan (University of Ottawa), Naveen Haribabu, and Dr. Julien Yockell-Lelievre (Ottawa Hospital Research Institute) for their assistance with the reverse kinase screen, Dr. William Stanford and Rina Al-awar (Ontario Institute for Cancer Research) for supplying the kinase inhibitor library, Serge Neault (University of Ottawa) for his expertise with the measles virus, and Dr. Guy Ungerechts (Ottawa Hospital Research Institute) for his generous provision of the measles virus for use in this study. We also thank Dr. Debbie C. Crans (Colorado State University) for helpful discussions surrounding vanadium chemistry.

J.S.D. is supported by the Terry Fox Research Institute (TFF 122868), the Canadian Institutes of Health Research (CIHR, INI-147824 and grant #705952), and the Canadian Cancer Society supported by the Lotte & John Hecht Memorial Foundation (703014). B.W. is supported by the Ontario Graduate Scholarships (OGS) and CIHR Doctoral Fellowship (CGS-D). A.B was supported by the Hans K. Uhthoff Graduate Fellowship and Queen Elizabeth II Graduate Scholarships in Science and Technology (QEII-GSST).

AUTHOR CONTRIBUTIONS

Conceptualization, B.W., A.B., and J.S.D.; Methodology, B.W., A.B., and J.S.D.; Investigation, B.W., A.B., R.A., N.A., G.M. and A.C.; Writing – Original Draft, B.W., R.A., and J.S.D.; Writing – Review & Editing, B.W., A.B., R.A., and J.S.D.; Funding Acquisition, J.S.D.; Supervision, R.A. and J.S.D.

DECLARATION OF INTERESTS

J.S.D is co-inventor on a patent covering the use of vanadium compounds as enhancer of RNA viruses and holds shares in Virica Biotech and Virano Therapeutics that holds a license to this and related intellectual property.

REFERENCES

1. Lichty, B.D., Breitbach, C.J., Stojdl, D.F., and Bell, J.C. (2014). Going viral with cancer immunotherapy. Nat. Rev. Cancer *14*, 559–567. https://doi.org/10.1038/nrc3770.

2. Pikor, L.A., Bell, J.C., and Diallo, J.-S. (2015). Oncolytic viruses: exploiting cancer's deal with the devil. Trends Cancer *1*, 266–277. https://doi.org/10.1016/j.trecan.2015.10.004.

3. Ilkow, C.S., Swift, S.L., Bell, J.C., and Diallo, J.-S. (2014). From scourge to cure: tumour-selective viral pathogenesis as a new strategy against cancer. PLoS Pathog. *10*, e1003836. https://doi.org/10.1371/journal.ppat.1003836.

4. Parato, K.A., Senger, D., Forsyth, P.A.J., and Bell, J.C. (2005). Recent progress in the battle between oncolytic viruses and tumours. Nat. Rev. Cancer *5*, 965–976. https://doi.org/10.1038/nrc1750.

5. Lawler, S.E., Speranza, M.-C., Cho, C.-F., and Chiocca, E.A. (2017). Oncolytic viruses in cancer treatment: a Review. JAMA Oncol. *3*, 841. https://doi.org/10.1001/jamaoncol.2016.2064.

6. Pol, J., Kroemer, G., and Galluzzi, L. (2016). First oncolytic virus approved for melanoma immunotherapy. OncoImmunology *5*, e1115641. https://doi.org/10.1080/2162402x.2015.1115641.

7. Rehman, H., Silk, A.W., Kane, M.P., and Kaufman, H.L. (2016). Into the clinic: talimogene laherparepvec (T-VEC), a first-in-class intratumoral oncolytic viral therapy. J. Immunother. Cancer *4*, 53. https://doi.org/10.1186/s40425-016-0158-5.

8. Russell, S.J., Peng, K.-W., and Bell, J.C. (2012). Oncolytic virotherapy. Nat. Biotechnol. *30*, 658–670. https://doi.org/10.1038/nbt.2287.

9. Liu, Y.-P., Suksanpaisan, L., Steele, M.B., Russell, S.J., and Peng, K.-W. (2013). Induction of antiviral genes by the tumor microenvironment confers resistance to virotherapy. Sci. Rep. *3*, 2375. https://doi.org/10.1038/srep02375.

10. Moerdyk-Schauwecker, M., Shah, N.R., Murphy, A.M., Hastie, E., Mukherjee, P., and Grdzelishvili, V.Z. (2013). Resistance of pancreatic cancer cells to oncolytic vesicular stomatitis virus: role of type I interferon signaling. Virology *436*, 221–234. https://doi.org/10.1016/j.virol.2012.11.014.

11. Diallo, J.-S., Boeuf, F.L., Lai, F., Cox, J., Vaha-Koskela, M., Abdelbary, H., MacTavish, H., Waite, K., Falls, T., Wang, J., et al. (2010). A high-throughput pharmacoviral approach identifies novel oncolytic virus sensitizers. Mol. Ther. *18*, 1123–1129. https://doi.org/10.1038/mt.2010.67.

12. Forbes, N.E., Krishnan, R., and Diallo, J.-S. (2014). Pharmacological modulation of anti-tumor immunity induced by oncolytic viruses. Front. Oncol. *4*, 191. https://doi.org/10.3389/fonc.2014.00191.

13. Dornan, M.H., Krishnan, R., Macklin, A.M., Selman, M., El Sayes, N., Son, H.H., Davis, C., Chen, A., Keillor, K., Le, P.J., et al. (2016). First-in-class small molecule potentiators of cancer virotherapy. Sci. Rep. *6*, 26786. https://doi.org/10.1038/srep26786.

14. Phan, M., Watson, M.F., Alain, T., and Diallo, J.-S. (2018). Oncolytic viruses on drugs: achieving higher therapeutic efficacy. ACS Infect. Dis. *4*, 1448–1467. https://doi.org/10.1021/acsinfecdis.8b00144.

15. Selman, M., Rousso, C., Bergeron, A., Son, H.H., Krishnan, R., El-Sayes, N.A., Varette, O., Chen, A., Le Boeuf, F., Tzelepis, F., et al. (2018). Multi-modal potentiation of oncolytic virotherapy by vanadium compounds. Mol. Ther. *26*, 56–69. https://doi.org/10.1016/j.ymthe.2017.10.014.

16. Bergeron, A., Kostenkova, K., Selman, M., Murakami, H.A., Owens, E., Haribabu, N., Arulanandam, R., Diallo, J.-S., and Crans, D.C. (2019). Enhancement of oncolytic virotherapy by vanadium(V) dipicolinates. Biometals Int. J. Role Met. Ions Biol. Biochem. Med. *32*, 545–561. https://doi.org/10.1007/s10534-019-00200-9.

17. McLauchlan, C.C., Peters, B.J., Willsky, G.R., and Crans, D.C. (2015). Vanadium–phosphatase complexes: phosphatase inhibitors favor the trigonal bipyramidal transition state geometries. Coord. Chem. Rev. *301–302*, 163–199. https://doi.org/10.1016/j.ccr.2014.12.012.

18. Irving, Elsa, and Stoker, Andrew (2017). Vanadium compounds as PTP inhibitors. Molecules *22*, 2269. https://doi.org/10.3390/molecules22122269.

19. Evangelou, A.M. (2002). Vanadium in cancer treatment. Crit. Rev. Oncol. Hematol. *42*, 249–265. https://doi.org/10.1016/s1040-8428(01)00221-9.

20. Garcia, V., Krishnan, R., Davis, C., Batenchuk, C., Le Boeuf, F., Abdelbary, H., and Diallo, J.-S. (2014). High-throughput titration of luciferase-expressing recombinant viruses. J. Vis. Exp. 51890. https://doi.org/10.3791/51890.

21. Herbst, R.S. (2004). Review of epidermal growth factor receptor biology. Int. J. Radiat. Oncol. *59*, S21–S26. https://doi.org/10.1016/j.ijrobp.2003.11.041.

22. Platanias, L.C. (2005). Mechanisms of type-I- and type-II-interferon-mediated signalling. Nat. Rev. Immunol. *5*, 375–386. https://doi.org/10.1038/nri1604.

23. Lupberger, J., Duong, F.H.T., Fofana, I., Zona, L., Xiao, F., Thumann, C., Durand, S.C., Pessaux, P., Zeisel, M.B., Heim, M.H., and Baumert, T.F. (2013). Epidermal growth factor receptor signaling impairs the antiviral activity of interferon-alpha: Hepatology. Hepatology *58*, 1225–1235. https://doi.org/10.1002/hep.26404.

24. Quesnelle, K.M., Boehm, A.L., and Grandis, J.R. (2007). STAT-mediated EGFR signaling in cancer. J. Cell. Biochem. *102*, 311–319. https://doi.org/10.1002/jcb.21475.

25. Essaghir, A., Toffalini, F., Knoops, L., Kallin, A., van Helden, J., and Demoulin, J.-B. (2010). Transcription factor regulation can be accurately predicted from the presence of target gene signatures in microarray gene expression data. Nucleic Acids Res. *38*, e120. https://doi.org/10.1093/nar/gkq149.

26. Brown, K., Park, S., Kanno, T., Franzoso, G., and Siebenlist, U. (1993). Mutual regulation of the transcriptional activator NF-kappa B and its inhibitor, I kappa B-alpha. Proc. Natl. Acad. Sci. *90*, 2532–2536. https://doi.org/10.1073/pnas.90.6.2532.

27. Beg, A.A., Ruben, S.M., Scheinman, R.I., Haskill, S., Rosen, C.A., and Baldwin, A.S. (1992). I kappa B interacts with the nuclear localization sequences of the subunits of NF-kappa B: a mechanism for cytoplasmic retention. Genes Dev. *6*, 1899–1913. https://doi.org/10.1101/gad.6.10.1899.

28. Sun, L., and Carpenter, G. (1998). Epidermal growth factor activation of NF-κB is mediated through IκBα degradation and intracellular free calcium. Oncogene *16*, 2095–2102. https://doi.org/10.1038/sj.onc.1201731.

29. Shostak, K., and Chariot, A. (2015). EGFR and NF-κB: partners in cancer. Trends Mol. Med. *21*, 385–393. https://doi.org/10.1016/j.molmed.2015.04.001.

30. Lawrence, T. (2009). The nuclear factor NF- B pathway in inflammation. Cold Spring Harb. Perspect. Biol. *1*, a001651. https://doi.org/10.1101/cshperspect.a001651.

31. Hiroi, M., and Ohmori, Y. (2003). The transcriptional coactivator CREB-binding protein cooperates with STAT1 and NF-κB for synergistic transcriptional activation of the CXC ligand 9/Monokine induced by interferon-γ gene. J. Biol. Chem. *278*, 651–660. https://doi.org/10.1074/jbc.m204544200.

32. Clarke, D.L., Clifford, R.L., Jindarat, S., Proud, D., Pang, L., Belvisi, M., and Knox, A.J. (2010). TNFα and IFNγ synergistically enhance transcriptional activation of CXCL10 in human airway smooth muscle cells via STAT-1, NF-κB, and the transcriptional coactivator CREB-binding protein. J. Biol. Chem. *285*, 29101–29110. https://doi.org/10.1074/jbc.m109.099952.

33. McAusland, T.M., van Vloten, J.P., Santry, L.A., Guilleman, M.M., Rghei, A.D., Ferreira, E.M., Ingrao, J.C., Arulanandam, R., Major, P.P., Susta, L., et al. (2021). Combining vanadyl sulfate with Newcastle disease virus potentiates rapid innate immune-mediated regression with curative potential in murine cancer models. Mol. Ther. - Oncolytics *20*, 306–324. https://doi.org/10.1016/j.omto.2021.01.009.

34. Hoang, H.-D., Graber, T.E., and Alain, T. (2018). Battling for ribosomes: translational control at the forefront of the antiviral response. J. Mol. Biol. *430*, 1965–1992. https://doi.org/10.1016/j.jmb.2018.04.040.

35. Thompson, K.H., and Orvig, C. (2006). Vanadium in diabetes: 100 years from phase 0 to phase I. J. Inorg. Biochem. *100*, 1925–1935. https://doi.org/10.1016/j.jinorgbio.2006.08.016.

36. Rozzo, C., Sanna, D., Garribba, E., Serra, M., Cantara, A., Palmieri, G., and Pisano, M. (2017). Antitumoral effect of vanadium compounds in malignant melanoma cell lines. J. Inorg. Biochem. *174*, 14–24. https://doi.org/10.1016/j.jinorgbio.2017.05.010.

37. Naso, L.G., Badiola, I., Marquez Clavijo, J., Valcarcel, M., Salado, C., Ferrer, E.G., and Williams, P.A.M. (2016). Inhibition of the metastatic progression of breast and colorectal cancer *in vitro* and *in vivo* in murine model by the oxidovanadium(IV) complex with luteolin. Bioorg. Med. Chem. *24*, 6004–6011. https://doi.org/10.1016/j.bmc.2016.09.058.

38. Wu, Y., Ma, Y., Xu, Z., Wang, D., Zhao, B., Pan, H., Wang, J., Xu, D., Zhao, X., Pan, S., et al. (2014). Sodium orthovanadate inhibits growth of human hepatocellular carcinoma cells *in vitro* and in an orthotopic model *in vivo*. Cancer Lett. *351*, 108–116. https://doi.org/10.1016/j.canlet.2014.05.018.

39. Bishayee, A., Waghray, A., Patel, M.A., and Chatterjee, M. (2010). Vanadium in the detection, prevention and treatment of cancer: the *in vivo* evidence. Cancer Lett. *294*, 1–12. https://doi.org/10.1016/j.canlet.2010.01.030.

40. Kim, Y.-R., Cha, H.-Y., Lim, K., Hwang, B.-D., Hoe, K.-L., Namgung, U., and Park, S.-K. (2003). Activation of epidermal growth factor receptor is responsible for pervanadate-induced phospholipase D activation. Exp. Mol. Med. *35*, 118–124. https://doi.org/10.1038/emm.2003.17.

41. Lahusen, T., Fereshteh, M., Oh, A., Wellstein, A., and Riegel, A.T. (2007). Epidermal growth factor receptor tyrosine phosphorylation and signaling controlled by a nuclear receptor coactivator, amplified in breast cancer 1. Cancer Res. *67*, 7256–7265. https://doi.org/10.1158/0008-5472.can-07-1013.

42. Wieduwilt, M.J., and Moasser, M.M. (2008). The epidermal growth factor receptor family: biology driving targeted therapeutics. Cell. Mol. Life Sci. *65*, 1566–1584. https://doi.org/10.1007/s00018-008-7440-8.

43. Uchida, H., Marzulli, M., Nakano, K., Goins, W.F., Chan, J., Hong, C.-S., Mazzacurati, L., Yoo, J.Y., Haseley, A., Nakashima, H., et al. (2013). Effective treatment of an orthotopic xenograft model of human glioblastoma using an EGFR-retargeted oncolytic herpes simplex virus. Mol. Ther. *21*, 561–569. https://doi.org/10.1038/mt.2012.211.

44. Piao, Y., Jiang, H., Alemany, R., Krasnykh, V., Marini, F.C., Xu, J., Alonso, M.M., Conrad, C.A., Aldape, K.D., Gomez-Manzano, C., and Fueyo, J. (2009). Oncolytic adenovirus retargeted to Delta-EGFR induces selective antiglioma activity. Cancer Gene Ther. *16*, 256–265. https://doi.org/10.1038/cgt.2008.75.

45. Zaoui, K., Bossow, S., Grossardt, C., Leber, M.F., Springfeld, C., Plinkert, P.K., von Kalle, C., and Ungerechts, G. (2012). Chemovirotherapy for head and neck squamous cell carcinoma with EGFR-targeted and CD/UPRT-armed oncolytic measles virus. Cancer Gene Ther. *19*, 181–191. https://doi.org/10.1038/cgt.2011.75.

46. Ueki, I.F., Min-Oo, G., Kalinowski, A., Ballon-Landa, E., Lanier, L.L., Nadel, J.A., and Koff, J.L. (2013). Respiratory virus–induced EGFR activation suppresses IRF1-dependent interferon λ and antiviral defense in airway epithelium. J. Exp. Med. *210*, 1929–1936. https://doi.org/10.1084/jem.20121401.

47. Mitchell, H.D., Eisfeld, A.J., Stratton, K.G., Heller, N.C., Bramer, L.M., Wen, J., McDermott, J.E., Gralinski, L.E., Sims, A.C., Le, M.Q., et al. (2019). The role of EGFR in influenza pathogenicity: multiple network-based approaches to identify a key regulator of non-lethal infections. Front. Cell Dev. Biol. *7*, 200. https://doi.org/10.3389/fcell.2019.00200.

48. Yang, L., Xu, J., Guo, L., Guo, T., Zhang, L., Feng, L., Chen, H., and Wang, Y. (2018). Porcine epidemic diarrhea virus-induced epidermal growth factor receptor activation impairs the antiviral activity of type I interferon. J. Virol. *92*. e02095–17. https://doi.org/10.1128/jvi.02095-17.

49. Diao, J., Pantua, H., Ngu, H., Komuves, L., Diehl, L., Schaefer, G., and Kapadia, S.B. (2012). Hepatitis C virus induces epidermal growth factor receptor activation via CD81 binding for viral internalization and entry. J. Virol. *86*, 10935–10949. https://doi.org/10.1128/jvi.00750-12.

50. Beerli, C., Yakimovich, A., Kilcher, S., Reynoso, G.V., Fläschner, G., Müller, D.J., Hickman, H.D., and Mercer, J. (2019). Vaccinia virus hijacks EGFR signalling to enhance virus spread through rapid and directed infected cell motility. Nat. Microbiol. *4*, 216–225. https://doi.org/10.1038/s41564-018-0288-2.

51. Wang, C., Wang, X., Veleeparambil, M., Kessler, P.M., Willard, B., Chattopadhyay, S., and Sen, G.C. (2020). EGFR-mediated tyrosine phosphorylation of STING determines its trafficking route and cellular innate immunity functions. EMBO J. *39*, e104106. https://doi.org/10.15252/embj.2019104106.

52. Lulli, D., Carbone, M.L., and Pastore, S. (2016). Epidermal growth factor receptor inhibitors trigger a type I interferon response in human skin. Oncotarget *7*, 47777–47793. https://doi.org/10.18632/oncotarget.10013.

53. Gong, K., Guo, G., Panchani, N., Bender, M.E., Gerber, D.E., Minna, J.D., Fattah, F., Gao, B., Peyton, M., Kernstine, K., et al. (2020). EGFR inhibition triggers an adaptive response by co-opting antiviral signaling pathways in lung cancer. Nat. Cancer *1*, 394–409. https://doi.org/10.1038/s43018-020-0048-0.

54. Qureshi, S.A., Leung, S., Kerr, I.M., Stark, G.R., and Darnell, J.E. (1996). Function of Stat2 protein in transcriptional activation by alpha interferon. Mol. Cell. Biol. *16*, 288–293. https://doi.org/10.1128/mcb.16.1.288.

55. Blaszczyk, K., Nowicka, H., Kostyrko, K., Antonczyk, A., Wesoly, J., and Bluyssen, H.A.R. (2016). The unique role of STAT2 in constitutive and IFN-induced transcription and antiviral responses. Cytokine Growth Factor Rev. *29*, 71–81. https://doi.org/10.1016/j.cytogfr.2016.02.010.

56. Park, C., Li, S., Cha, E., and Schindler, C. (2000). Immune response in Stat2 knockout mice. Immunity *13*, 795–804. https://doi.org/10.1016/s1074-7613(00)00077-7.

57. Christian, S.L., Collier, T.W., Zu, D., Licursi, M., Hough, C.M., and Hirasawa, K. (2009). Activated Ras/MEK inhibits the antiviral response of alpha interferon by reducing STAT2 levels. J. Virol. *83*, 6717–6726. https://doi.org/10.1128/jvi.02213-08.

58. Noser, J.A., Mael, A.A., Sakuma, R., Ohmine, S., Marcato, P., WK Lee, P., and Ikeda, Y. (2007). The RAS/Raf1/MEK/ERK signaling pathway facilitates VSV-mediated oncolysis: implication for the defective interferon response in cancer cells. Mol. Ther. *15*, 1531–1536. https://doi.org/10.1038/sj.mt.6300193.

59. Han, W., Carpenter, R.L., Cao, X., and Lo, H.-W. (2013). STAT1 gene expression is enhanced by nuclear EGFR and HER2 via cooperation with STAT3: STAT1 regulation BY nuclear EGFR and STAT3. Mol. Carcinog. *52*, 959–969. https://doi.org/10.1002/mc.21936.

60. Andersen, P., Pedersen, M.W., Woetmann, A., Villingshøj, M., Stockhausen, M.-T., Ødum, N., and Poulsen, H.S. (2008). EGFR induces expression of IRF-1 via STAT1 and STAT3 activation leading to growth arrest of human cancer cells: EGFR Mediated IRF-1 Expression through STAT Activation. Int. J. Cancer *122*, 342–349. https://doi.org/10.1002/ijc.23109.

61. Cheng, C., Lin, H., Tsai, K., Chiang, Y., Lim, K., Chen, C.G., Su, Y., Peng, C., Ho, A., Huang, L., et al. (2018). Epidermal growth factor induces STAT1 expression to exacerbate the IFNr-mediated PD-L1 axis in epidermal growth factor receptor-positive cancers. Mol. Carcinog. *57*, 1588–1598. https://doi.org/10.1002/mc.22881.

62. Wu, T.R., Hong, Y.K., Wang, X.-D., Ling, M.Y., Dragoi, A.M., Chung, A.S., Campbell, A.G., Han, Z.-Y., Feng, G.-S., and Chin, Y.E. (2002). SHP-2 is a dual-specificity phosphatase involved in Stat1 dephosphorylation at both tyrosine and serine residues in nuclei. J. Biol. Chem. *277*, 47572–47580. https://doi.org/10.1074/jbc.m207536200.

63. Wang, S., Zheng, G., Zhao, L., Xu, F., and Qian, J. (2015). Shp-2 contributes to anti-RSV activity in human pulmonary alveolar epithelial cells by interfering with the IFN-α-induced Jak/Stat1 pathway. J. Cell. Mol. Med. *19*, 2432–2440. https://doi.org/10.1111/jcmm.12629.

64. Wang, Q., Pan, W., Wang, S., Pan, C., Ning, H., Huang, S., Chiu, S.-H., and Chen, J.-L. (2021). Protein tyrosine phosphatase SHP2 suppresses host innate immunity against influenza A virus by regulating EGFR-mediated signaling. J. Virol. *95*, e02001–e02020. /jvi/95/6/JVI.02001-20.atom. https://doi.org/10.1128/jvi.02001-20.

65. Selman, M., Ou, P., Rousso, C., Bergeron, A., Krishnan, R., Pikor, L., Chen, A., Keller, B.A., Ilkow, C., Bell, J.C., and Diallo, J.S. (2018). Dimethyl fumarate potentiates oncolytic virotherapy through NF-κB inhibition. Sci. Transl. Med. *10*, eaao1613. https://doi.org/10.1126/scitranslmed.aao1613.

66. Du, Z., Whitt, M.A., Baumann, J., Garner, J.M., Morton, C.L., Davidoff, A.M., and Pfeffer, L.M. (2012). Inhibition of type I interferon-mediated antiviral action in human glioma cells by the IKK inhibitors BMS-345541 and TPCA-1. J. Interferon Cytokine Res. *32*, 368–377. https://doi.org/10.1089/jir.2012.0002.

67. Cataldi, M., Shah, N.R., Felt, S.A., and Grdzelishvili, V.Z. (2015). Breaking resistance of pancreatic cancer cells to an attenuated vesicular stomatitis virus through a novel activity of IKK inhibitor TPCA-1. Virology *485*, 340–354. https://doi.org/10.1016/j.virol.2015.08.003.

68. Ivashkiv, L.B., and Donlin, L.T. (2014). Regulation of type I interferon responses. Nat. Rev. Immunol. *14*, 36–49. https://doi.org/10.1038/nri3581.

69. Ohmori, Y., Schreiber, R.D., and Hamilton, T.A. (1997). Synergy between interferon-γ and tumor necrosis factor-α in transcriptional activation is mediated by cooperation between signal transducer and activator of transcription 1 and nuclear factor κB. J. Biol. Chem. *272*, 14899–14907. https://doi.org/10.1074/jbc.272.23.14899.

70. Shelton, J.G., Steelman, L.S., Abrams, S.L., Bertrand, F.E., Franklin, R.A., McMahon, M., and McCubrey, J.A. (2005). The epidermal growth factor receptor gene family as a target for therapeutic intervention in numerous cancers: what's genetics got to do with it? Expert Opin. Ther. Targets *9*, 1009–1030. https://doi.org/10.1517/14728222.9.5.1009.

71. Sigismund, S., Avanzato, D., and Lanzetti, L. (2018). Emerging functions of the EGFR in cancer. Mol. Oncol. *12*, 3–20. https://doi.org/10.1002/1878-0261.12155.

72. Jutten, B., Keulers, T.G., Schaaf, M.B.E., Savelkouls, K., Theys, J., Span, P.N., Vooijs, M.A., Bussink, J., and Rouschop, K.M.A. (2013). EGFR overexpressing cells and tumors are dependent on autophagy for growth and survival. Radiother. Oncol. *108*, 479–483. https://doi.org/10.1016/j.radonc.2013.06.033.

73. Liccardi, G., Hartley, J.A., and Hochhauser, D. (2011). EGFR nuclear translocation modulates DNA repair following cisplatin and ionizing radiation treatment. Cancer Res. *71*, 1103–1114. https://doi.org/10.1158/0008-5472.can-10-2384.

74. Tao, Y., Song, X., Deng, X., Xie, D., Lee, L.M., Liu, Y., Li, W., Li, L., Deng, L., Wu, Q., et al. (2005). Nuclear accumulation of epidermal growth factor receptor and acceleration of G1/S stage by Epstein–Barr-encoded oncoprotein latent membrane protein 1. Exp. Cell Res. *303*, 240–251. https://doi.org/10.1016/j.yexcr.2004.09.030.

75. Arulanandam, R., Batenchuk, C., Angarita, F.A., Ottolino-Perry, K., Cousineau, S., Mottashed, A., Burgess, E., Falls, T.J., De Silva, N., Tsang, J., et al. (2015).

VEGF-mediated induction of PRD1-BF1/Blimp1 expression sensitizes tumor vasculature to oncolytic virus infection. Cancer Cell *28*, 210–224. https://doi.org/10.1016/j.ccell.2015.06.009.

76. Stojdl, D.F., Lichty, B.D., tenOever, B.R., Paterson, J.M., Power, A.T., Knowles, S., Marius, R., Reynard, J., Poliquin, L., Atkins, H., et al. (2003). VSV strains with defects in their ability to shutdown innate immunity are potent systemic anti-cancer agents. Cancer Cell *4*, 263–275. https://doi.org/10.1016/s1535-6108(03)00241-1.

77. Diallo, J.-S., Vähä-Koskela, M., Le Boeuf, F., and Bell, J. (2012). Propagation, purification, and *in vivo* testing of oncolytic vesicular stomatitis virus strains. In Oncolytic Viruses Methods in Molecular Biology, D.H. Kirn, T.-C. Liu, and S.H. Thorne, eds. (Humana Press), pp. 127–140.

OMTO, Volume *25*

Supplemental information

Dependency of EGFR activation in vanadium-based sensitization to oncolytic virotherapy

Boaz Wong, Anabel Bergeron, Nouf Alluqmani, Glib Maznyi, Andrew Chen, Rozanne Arulanandam, and Jean-Simon Diallo

Table S1: Key Resources Table

REAGENT or RESOURCE	SOURCE	IDENTIFIER
Antibodies		
Phosphorylated STAT1 (Tyr701), rabbit	Cell Signaling Technology	7649
STAT1, rabbit	Cell Signaling Technology	9172
Phosphorylated STAT2 (Tyr690), rabbit	Cell Signaling Technology	88410
STAT2, rabbit	Cell Signaling Technology	72604
Phosphorylated EGFR (Tyr1068), rabbit	Cell Signaling Technology	2234
EGFR, rabbit	Cell Signaling Technology	4267
Phosphorylated NF-κB/p65 (Ser536), rabbit	Cell Signaling Technology	3033
NF-κB/p65, rabbit	Cell Signaling Technology	8242
IκB-α (Amino-terminal antigen), mouse	Cell Signaling Technology	4814
Lamin B1, rabbit	Cell Signaling Technology	12586
α-tubulin, mouse	Santa Cruz	sc-8035
β-actin, rabbit	Cell Signaling Technology	4970
hFAB™ Rhodamine Anti-Actin	Bio-Rad	12004163
hFAB™ Rhodamine Anti-GAPDH	Bio-Rad	12004168
Anti-rabbit HRP Secondary Antibody	New England Biolabs	7074
Anti-rabbit IgG (H+L) Alexa Fluor® 555	Cell Signaling Technology	4413S
Bacterial and virus strains		
VSVΔ51-Green fluorescent protein (GFP)	Dr. Jean-Simon Diallo	
VSVΔ51ΔG-GFP	Dr. Jean-Simon Diallo	
VSVΔ51-Firefly luciferase (FLuc)	Dr. Jean-Simon Diallo	
Measles virus, Schwartz strain - GFP	Dr. Guy Ungerechts	
Chemicals, peptides, and recombinant proteins		
Gefitinib	BioVision	1589
Erlotinib	BioVision	2048
UO126	Sigma-Aldrich	662005
IMD-0354	Sigma-Aldrich	I3159

SC-514	Sigma-Aldrich	SML0557
Human EGF	R&D Systems	236-EG
Human EGFR (cetuximab) antibody	InvivoGen	hegfr-mab1
Human IFNβ	PBL Assay Science	11415-1
Critical commercial assays		
Human CXCL10/IP-10 DuoSet Assay kit	R&D Systems	DY266
Deposited Data		
All uncropped Western blot images and raw data	Mendeley Data	doi:10.17632/zzpvtnmdw7.1
Experimental models: Cell lines		
786-0, human renal cell carcinoma	ATCC	CRL-1932
CT26WT, murine colon carcinoma	ATCC	CRL-2638
Vero, African green monkey renal cells	ATCC	CRL-81
TC-1, murine lung carcinoma	Dr. Guy Ungerechts	
Experimental models: Organisms/strains		
Female Balb/c mice	Charles River Laboratories	
Oligonucleotides		
Please see supplementary table S2 for oligonucleotides	Thermo Scientific	
Software and algorithms		
Prism 9.0	GraphPad	

Table S2: List of primers used in this study.

**Model**	**Gene**	**Forward primer (5' to 3')**	**Reverse primer (5' to 3')**
VSV	M	ATACTCAGATGTGGCAGCCG	GATCTGCCAATACCGCTGGA
	N	CATGTCACTGCAAGGCCTAAGA	GGCAGTATCGTGAATTCGATGC-3
Human	STAT1	ATGGCAGTCTGGCGGCTGAATT	CCAAACCAGGCTGGCACAATTG
	STAT2	CAGGTCACAGAGTTGCTACAGC	CGGTGAACTTGCTGCCAGTCTT
	GAPDH	ACAGTCAGCCGCATCTTCTT	GTTAAAAGCAGCCCTGGTGA
	IFNβ	CATTACCTGAAGGCCAAGGA	CAGCATCTGCTGGTTGAAGA
	IL-1b	CCACAGACCTTCCAGGAGAATG	GTGCAGTTCAGTGATCGTACAGG
	TNF-α	GCTGCACTTTGGAGTGATCG	GAGGGTTTGCTACAACATGGG
	CCL5	GCAGTCGTCCACAGGTCAAG	TCTTCTCTGGGTTGGCACAC
	IL-6	ACCCCCAATAAATATAGGACTGGA	GAAGGCGCTTGTGGAGAAGG
	CXCL9	AGTGCAAGGAACCCCAGTAG	AGGGCTTGGGGCAAATTGTT
	CXCL10	CTGAGCCTACAGCAGAGGAAC	AGGTACTCCTTGAATGCCACTT

	MX2	GAACGTGCAGCGAGCTTGTC	AAGGCTTGTGGGCCTTAGAC
	IFITM1	CCGTGAAGTCTAGGGACAG G	GGTAGACTGTCACAGAGCCG
Mouse	GAPDH	CATCACTGCCACCCAGAAGA CTG	ATGCCAGTGAGCTTCCCGTT CAG
	CXCL9	CAGTGTGGAGTTCGAGGAAC C	TTTGTTGCAATTGGGGCTTG G
	CXCL10	ATCATCCCTGCGAGCCTATC CT	GACCTTTTTTGGCTAAACGC TTTC

S1

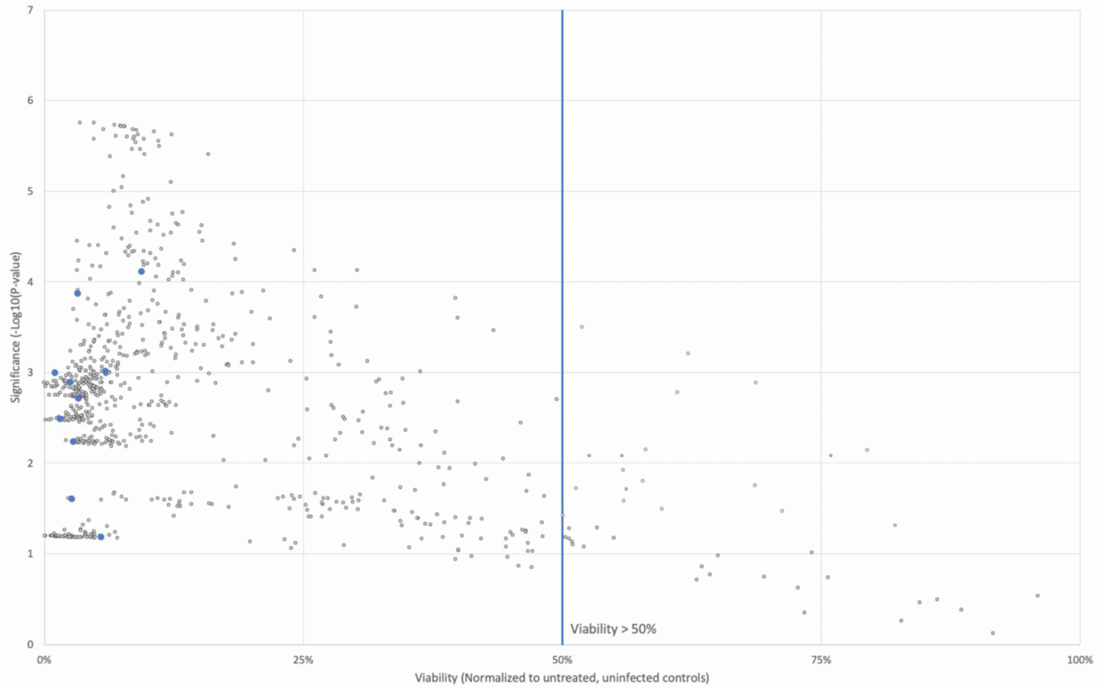

Significance (-Log10(P-value))
Viability > 50%
Viability (Normalized to untreated, uninfected controls)
0%
25%
50%
75%
100%

S2

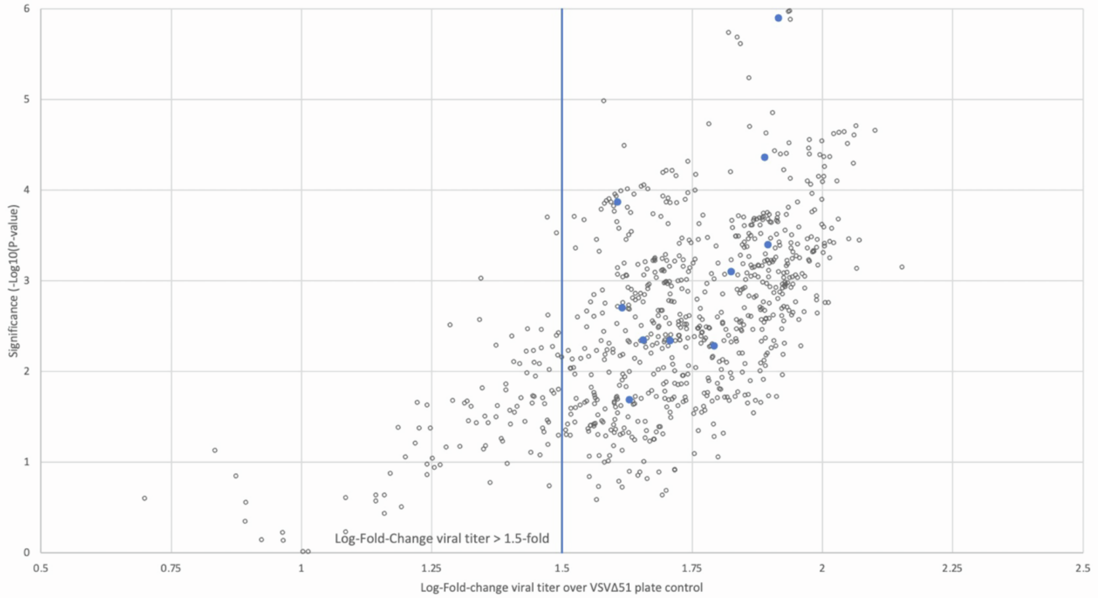

Significance (-Log10(P-value))
Log-Fold-Change viral titer > 1.5-fold
Log-Fold-change viral titer over VSVΔ51 plate control

Figure S1: Scatter plot of reverse kinase screen viability. 786-0 cells were pre-treated with a drug library of kinase inhibitors (1μM) and vanadate (125μM) for four hours and subsequently infected with VSVΔ51-FLuc at a MOI of 0.1. Vanadate controls are shown in blue. 48 hours post infection (hpi), the metabolic activity (surrogate for viability) was measured using resazurin (Alamar blue) assay. Values were blank-controlled then normalized against infected, untreated 786-0 (mock-infected) controls. An unpaired t-test assuming unequal variances was performed, the y-axis represents the calculated $-\text{Log}_{10}$(P-value) (n=2-3).

Figure S2: Scatter plot of reverse kinase screen vial titer. 48 hours post infection (hpi), the viral titer was determined using high-throughput virus assay. Values were normalized against infected, untreated 786-0 (mock-infected) controls. An unpaired t-test assuming unequal variances was performed, the y-axis represents the calculated $-\text{Log}_{10}$(P-value) (n=2-3).

S3

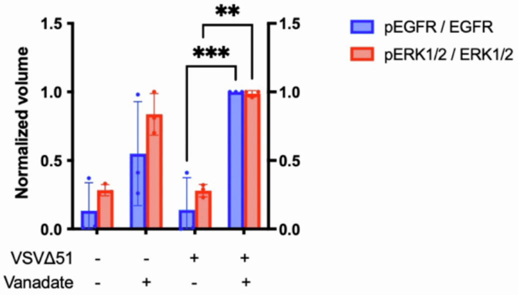
Normalized volume
1.5
1.0
0.5
0.0
**
***
pEGFR / EGFR
pERK1/2 / ERK1/2
VSVΔ51 - - + +
Vanadate - + - +

S4

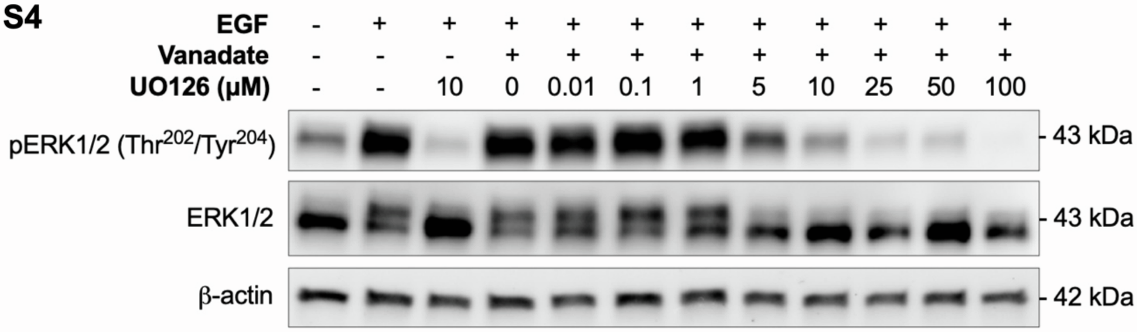
EGF - + + + + + + + + + + +
Vanadate - - - + + + + + + + + +
UO126 (μM) - - 10 0 0.01 0.1 1 5 10 25 50 100
pERK1/2 (Thr202/Tyr204)
ERK1/2
β-actin
43 kDa
43 kDa
42 kDa

S5

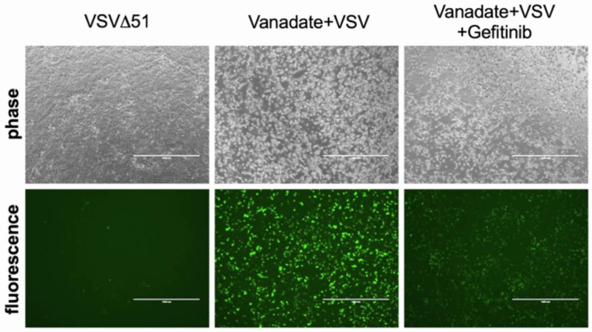
VSVΔ51
Vanadate+VSV
Vanadate+VSV
+Gefitinib
phase
fluorescence

S6

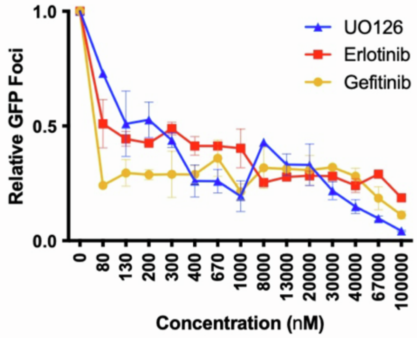
Relative GFP Foci
1.0
0.5
0.0
UO126
Erlotinib
Gefitinib
0
80
130
200
300
400
670
1000
8000
13000
20000
30000
40000
67000
100000
Concentration (nM)

S7

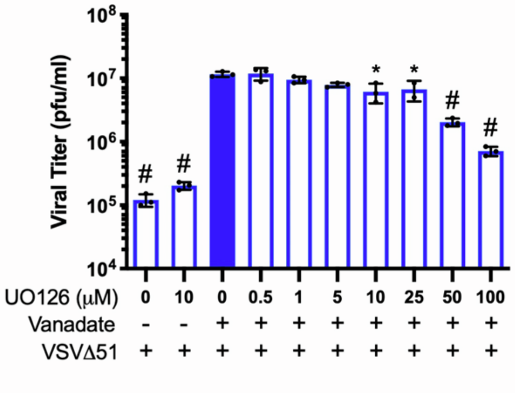
Viral Titer (pfu/ml)
10^8
10^7
10^6
10^5
10^4
#
#
*
*
#
#
UO126 (μM) 0 10 0 0.5 1 5 10 25 50 100
Vanadate - - + + + + + + + +
VSVΔ51 + + + + + + + + + +

Figure S3: pEGFR:EGFR and pERK1/2:ERK1/2 volume densitometry ratios corresponding to Figure 1D (n=3, mean ± SD).

Figure S4: Human 786-0 renal carcinoma cells were treated pre-treated with or without vanadate (150 μM) and varying concentrations of UO126 (0 – 100μM) as indicated for four hours. Cells were then treated with 10ng/mL EGF for 10 minutes and lysates were probed by western blot for phosphorylated ERK1/2, total ERK1/2 and β-actin.

Figure S5: Human 786-0 cells were pretreated ± vanadate (150μM) ± gefitinib (10μM) for 4 hours, then consequently infected with VSVΔ51-GFP (MOI 0.01). Phase and fluorescence images were taken 24 hours post infection (hpi); scale bar = 1000μm.

Figure S6: Count of GFP foci were obtained from Figure 2B and plotted relative to the vanadate + VSVΔ51 condition without kinase inhibitor (n=2, mean ± SEM).

Figure S7: Human 786-0 cells were pretreated ± vanadate (150μM) ± UO126 (0 – 100μM) for 4 hours, then consequently infected with VSVΔ51-GFP (MOI 0.01). Supernatants were collected 24hpi and titered by viral plaque assay (n=3, mean ± SD; *$P<0.05$, $^{\#}P<0.0001$; one-way ANOVA compared to the infected VSVΔ51 + vanadate only condition as indicated by the filled bar).

S8

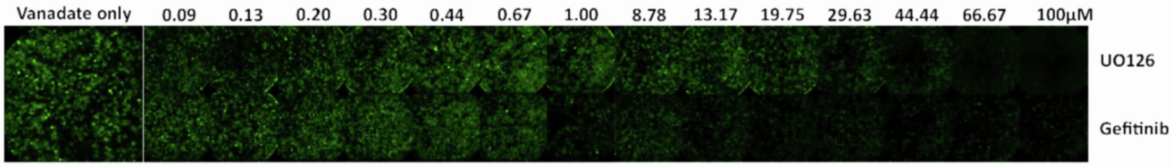
Vanadate only
0.09
0.13
0.20
0.30
0.44
0.67
1.00
8.78
13.17
19.75
29.63
44.44
66.67
100μM
UO126
Gefitinib

S9

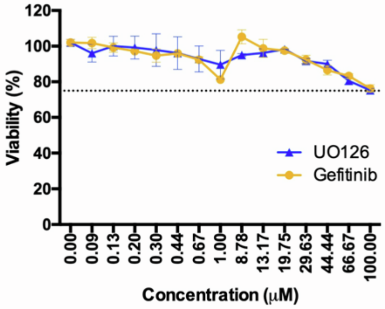
Viability (%)
120
100
80
60
40
20
0
0.00
0.09
0.13
0.20
0.30
0.44
0.67
1.00
8.78
13.17
19.75
29.63
44.44
66.67
100.00
Concentration (μM)
UO126
Gefitinib

S10

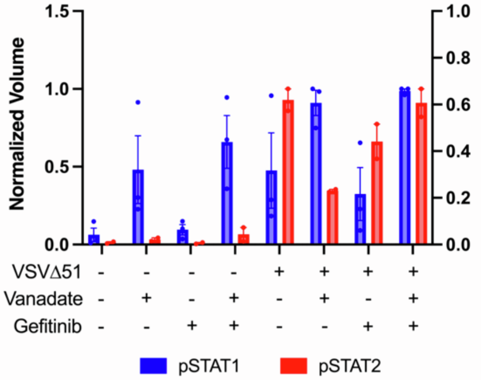
Normalized Volume
1.5
1.0
0.5
0.0
1.0
0.8
0.6
0.4
0.2
0.0
VSVΔ51 - - - - + + + +
Vanadate - + - + - + - +
Gefitinib - - + + - - + +
pSTAT1
pSTAT2

S11

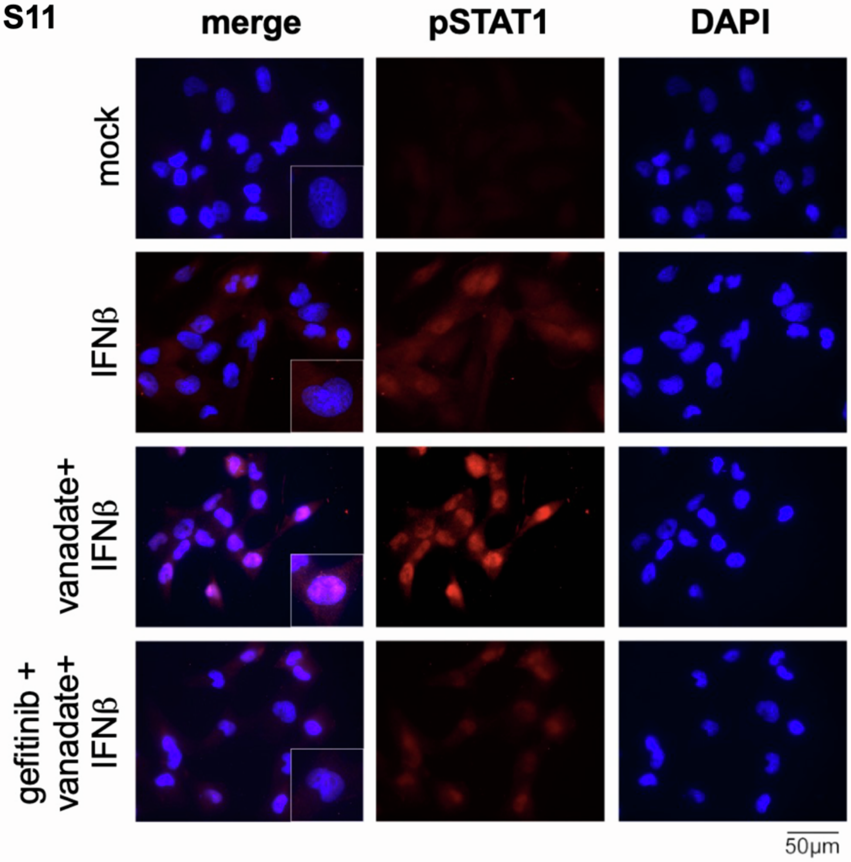
merge
pSTAT1
DAPI
mock
IFNβ
vanadate+ IFNβ
gefitinib + vanadate+ IFNβ
50μm

Figure S8: Human renal 786-0 cancer cells were simultaneously treated with varying concentrations of gefitinib or UO126 (0 - 100 μM) and vanadate (150 μM). Four hours later, cells were infected with measles virus expressing GFP (MOI 0.3). Representative fluorescent images were taken 24 hours post infection (hpi).

Figure S9: Cell viability was measured by Alamar blue assay and compared relative to the vanadate + VSVΔ51 condition without kinase inhibitor (n=2, mean ± SEM). Dotted line represents 75% viability.

Figure S10: Volume densitometry of pSTAT1 and pSTAT2 levels from Figure 3A (n=3, mean ± SD).

Figure S11: Representative immunofluorescence images corresponding to Figure 3D. Scale bar = 50μm.

S12

TF	*Pval*	*Eval*	∩	#TG	RC (%)
STAT1	< 1.0E-06	< 1.0E-06	8	25	0
NFKB1	4.00E-05	1.68E-03	7	63	0

S13

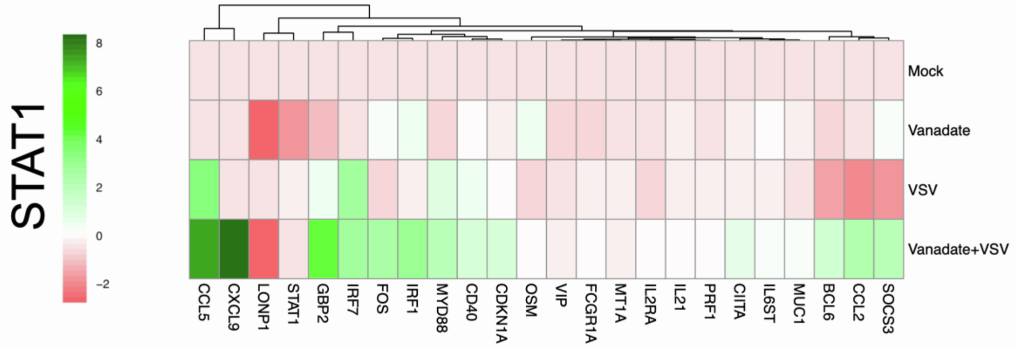

S14

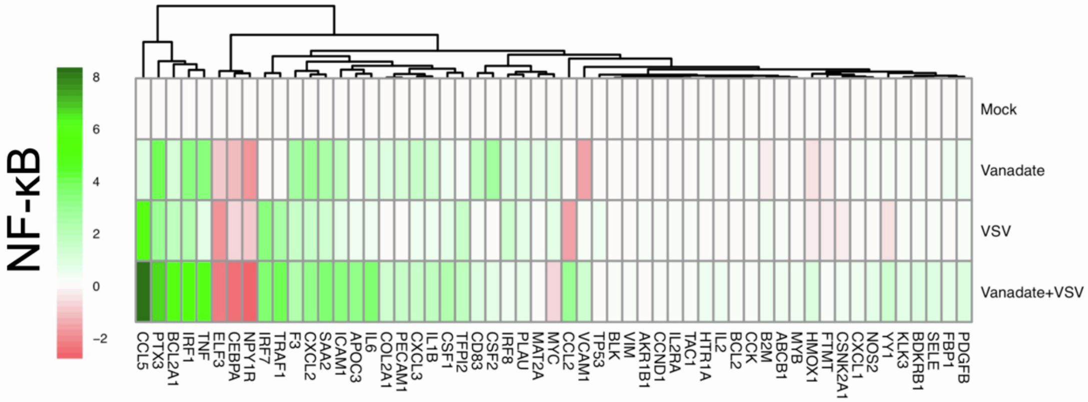

S15

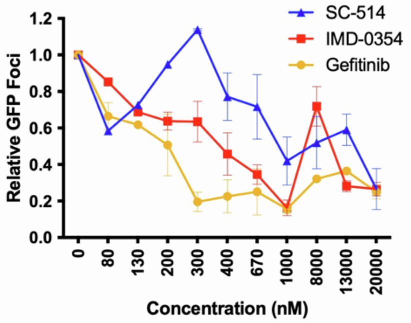

S16

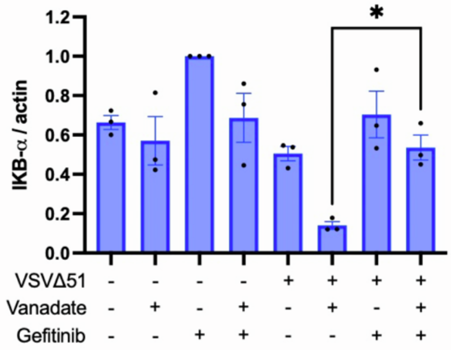

Figure S12: Table listing STAT1 and NF-κB transcription factors from the TFactS *in silico* analysis: *Pval* is P-value, *Eval* is E-value, ⋂ is the number of genes common between TF target genes and the query list, #TG is number of target genes for the corresponding TF and RC(%) is random control percentage which is a non-parametric control of false positives. Refer to published paper for more details on these parameters [29].

Figure S13: Heatmap of genes downstream to STAT1 transcription factor on 786-0 cells treated ± vanadate (150μM) and ±VSVΔ51 infection (MOI 0.01).

Figure S14: Heatmap of genes downstream to NF-κB transcription factor on 786-0 cells treated ± vanadate (150μM) and ±VSVΔ51 infection (MOI 0.01).

Figure S15: Count of GFP foci were obtained from Figure 4B and plotted relative to the vanadate + VSVΔ51 condition without kinase inhibitor (n=2, mean ± SEM).

Figure S16: Volume densitometry of IκB-α levels from Figure 4E (n=3, mean ± SD, *P<0.05 by one-way ANOVA).

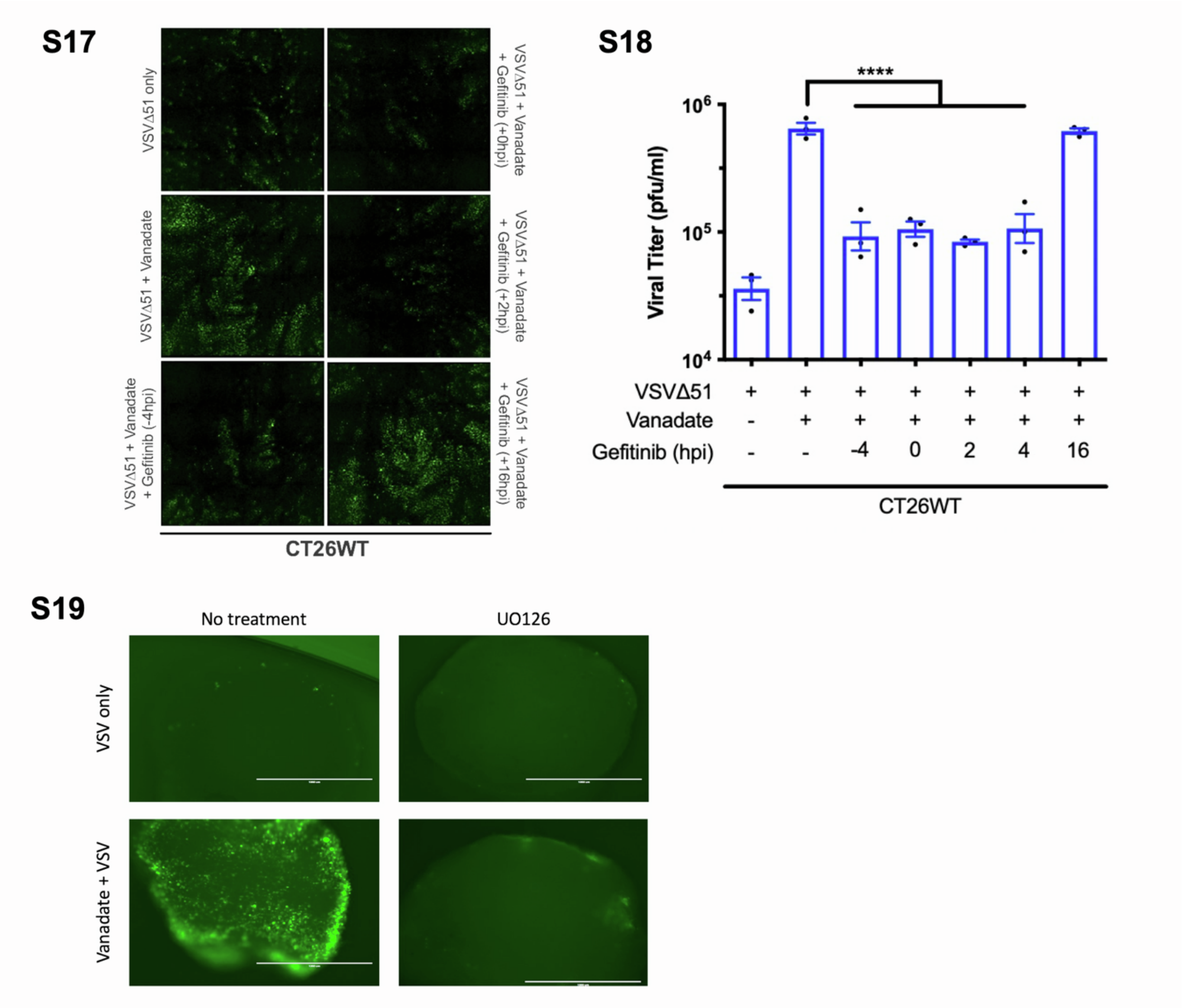

Figure S17: Mouse CT26WT colon carcinoma cells were pretreated ± vanadate (150µM) for 4 hours ± gefitinib (10µM) at -4, 0.5, 1, 2, 4 and 16 hours post infection (hpi), then consequently infected with VSVΔ51-GFP (MOI 0.01). Representative images are shown.

Figure S18: Supernatant from Figure S16 was collected 24hpi and titered by plaque assay (n=3, mean ± SD; ****P<0.0001 by one-way ANOVA).

Figure S19: *Ex vivo* mouse CT26WT tumors implanted subcutaneously in Balb/c mice were pretreated ± vanadate (150µM) ± UO126 (20µM) for 4 hours, then consequently infected with VSVΔ51-GFP (3e4 pfu/core). Representative fluorescence images were taken 24 hours post infection. Scale bar = 1000 µm.
